# Ultrasmall PtAu_2_ nanoclusters activate endogenous anti-inflammatory and anti-oxidative systems to prevent inflammatory osteolysis

**DOI:** 10.7150/thno.80514

**Published:** 2023-01-22

**Authors:** Xuzhuo Chen, Xiankun Cao, Dasheng Zheng, Chang Li, Yan Chen, Keyu Kong, Weifeng Xu, Bin Shi, Xinwei Chen, Fengrong Dai, Shanyong Zhang

**Affiliations:** 1 Department of Oral Surgery, Shanghai Key Laboratory of Stomatology & Shanghai Research Institute of Stomatology, National Clinical Research Center for Oral Diseases, Shanghai Ninth People's Hospital, College of Stomatology, Shanghai Jiao Tong University School of Medicine, Shanghai, 200011, China.; 2 State Key Laboratory of Structural Chemistry, Fujian Institute of Research on the Structure of Matter, Chinese Academy of Sciences, Fuzhou, Fujian, 350002, China.; 3 Department of Orthopedics, Shanghai Key Laboratory of Orthopedic Implant, Shanghai Ninth People's Hospital, Shanghai Jiao Tong University School of Medicine, Shanghai, 200011, China.; 4 Department of Oral and Maxillofacial Surgery, The First Affiliated Hospital, Fujian Medical University, Fuzhou 350002, Fujian, China.

**Keywords:** Metal cluster, Alloy cluster, Osteolysis, Osteoclast, Nrf2

## Abstract

**Rationale:** Inflammatory osteolysis, characterized by abundant immune cell infiltration and osteoclast (OC) formation, is a common complication induced by bacterial products and/or wear particles at the bone-prosthesis interface that severely reduces long-term stability after implantation. Molecular nanoclusters are ultrasmall particles with unique physicochemical and biological properties that have great potential as theranostic agents for treating inflammatory diseases.

**Methods:** In this study, heterometallic PtAu_2_ nanoclusters with sensitive nitric oxide-responsive phosphorescence turn-on characteristics and strong binding interactions with cysteine were designed, making them desirable candidates for the treatment of inflammatory osteolysis.

**Results:** PtAu_2_ clusters exhibited satisfactory biocompatibility and cellular uptake behavior, with potent anti-inflammatory and anti-OC activities *in vitro*. In addition, PtAu_2_ clusters alleviated lipopolysaccharide-induced calvarial osteolysis *in vivo* and activated nuclear factor erythroid 2-related factor 2 (Nrf2) expression by disrupting its association with Kelch-like ECH-associated protein 1 (Keap1), thereby upregulating the expression of endogenous anti-inflammatory and anti-oxidative products.

**Conclusion:** Through the rational design of novel heterometallic nanoclusters that activate the endogenous anti-inflammatory system, this study provides new insights into the development of multifunctional molecular therapeutic agents for inflammatory osteolysis and other inflammatory diseases.

## Introduction

Total joint replacement (TJR) is an effective surgical modality for treating end-stage joint pathologies such as osteoarthritis, rheumatoid arthritis, and traumatic arthritis 1, 2. Although TJR can improve articular morphology, function, pain relief, and quality of life, approximately 3-5% of patients experience postoperative aseptic loosening and periprosthetic infection 3. These complications can be induced by bacterial products and/or wear particles generated by prosthesis components, leading to inflammatory osteolysis 4. Acute/chronic inflammatory reactions and bone erosion at the bone-prosthesis interface caused by bacterial products such as lipopolysaccharides (LPS) can severely reduce the long-term stability of prostheses. Indeed, patients with inflammatory osteolysis may require multiple revision surgeries and even experience implant failure 5. In addition to surgical intervention, medications such as bisphosphonates and non-steroidal anti-inflammatory drugs (NSAIDs) are often required to prevent the progression of inflammation and bone erosion. However, over-administration can induce adverse side effects, making it highly important to investigate new curative strategies for periprosthetic inflammatory osteolysis 6.

Osteolysis commonly occurs around areas of inflammation and is characterized by the infiltration of abundant osteoclasts (OCs) and immune cells 7. As a principal source of inflammatory cytokines, macrophages play a central role in the pathogenesis of osteolysis and can differentiate into OCs, the main effector cells of osteolysis 8. Accumulating evidence suggests that inflammatory cytokines such as tumor necrosis factor-α (TNF-α), interleukin (IL)-1β, IL-6, and inducible nitric oxide synthase (iNOS) may stimulate OC formation, even in the absence of the receptor activator of NF-κB ligand (RANKL) 9-12. It has also been reported that RANKL and TNF-α exert synergistic effects on osteoclastogenesis, with low RANKL levels directly stimulating OC precursors to become mature OCs 13. IL-1β is another cytokine that is abundant in inflamed tissues and can enhance inflammatory osteolysis by cooperating with TNF-α to stimulate the expression of RANKL and its receptor, RANK 14. This clear interdependency between inflammatory cytokines and RANKL suggests the importance of blocking both the inflammatory reactions and osteoclastogenesis in an osteolytic environment.

In recent years, nanotherapies have attracted increasing attention because of their beneficial characteristics, including accurate targeting, responsive release, and flexible functionalization 15. Molecular nanoclusters are ultrasmall particles with core-shells of less than 3 nm 16. Unlike large nanoparticles, nanoclusters have precise molecular structures, uniform molecular sizes, good biocompatibility, facile synthesis, and promising biomedical applications 17-19. Nanocluster cores can be homometallic (e.g., Au, Ag, Cu, and Pt) or heterometallic with discrete energy levels, which can endow nanoclusters with unique chemical, biological, optical, and electronic properties 16. For example, Au clusters have been widely studied for the diagnosis and therapy of inflammation, cancer, implant-associated infections, and COVID-19 20-28, whereas heterometallic or alloy nanoclusters, such as Pt-Au clusters, have been reported to improve the physicochemical and biological performance of cancer therapies compared to homometallic nanoclusters 29, 30. The enhanced biomedical activity of heterometallic or alloy clusters is probably due to their improved stability and structural diversity resulting from the complementary advantages of different metal ions, which can favorably bind to proteins and enzymes and exert biological effects. Consequently, alloy nanoclusters have great potential as theranostic agents for treating inflammatory diseases.

**Figure 1:**
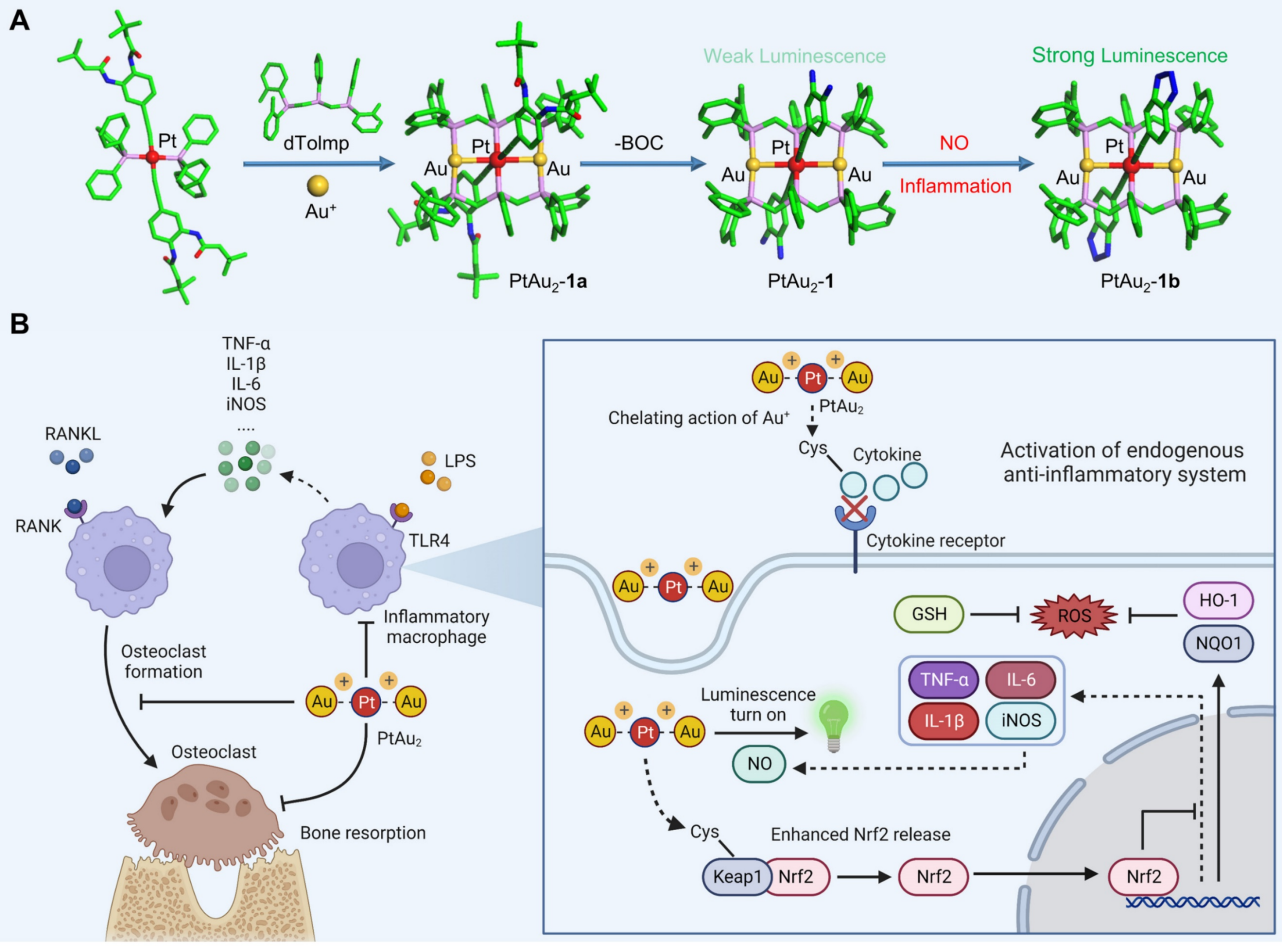
Schematic representation of the design, synthesis, and potential application of PtAu_2_ clusters to treat inflammatory osteolysis. **(A)** Synthesis of PtAu_2_ clusters.** (B)** Application of PtAu_2_ clusters to manage inflammatory osteolysis by disrupting the Keap1-Nrf2 association, increasing the release and nuclear translocation of Nrf2, and upregulating the expression of endogenous anti-inflammatory and anti-oxidative products (created with BioRender.com).

In this study, we designed a platinum(II)-gold(I) (PtAu_2_) nanocluster protected by bis(diphenylphosphinomethyl)phosphine as an effective anti-inflammatory and anti-osteolytic agent (Figure 1**A**). Owing to its d^8^-d^10^ heterometallic nature, the ultrasmall PtAu_2_ cluster (PtAu_2_-**1**) with electron-rich 4-ethynylbenzene-1,2-diamine acts as a highly-sensitive luminescence sensor in response to nitric oxide (NO), producing distinct phosphorescence turn-on characteristics that allow the detection of inflammation, as well as showing strong binding interactions with cysteine. In addition, the PtAu_2_ clusters exhibited satisfactory biocompatibility and cellular uptake behavior as well as potent anti-inflammatory and anti-OC effects* in vitro* and alleviated LPS-induced calvarial osteolysis *in vivo*. More importantly, PtAu_2_ clusters activated nuclear factor erythroid 2-related factor 2 (Nrf2) expression by disrupting its association with Kelch-like ECH-associated protein 1 (Keap1), thereby increasing the release and nuclear translocation of Nrf2, which upregulates the expression of endogenous anti-inflammatory and anti-oxidative products (Figure 1**B**). Unlike other reported nanoscale anti-inflammatory systems with complicated synthetic procedures, PtAu_2_ clusters can activate endogenous anti-oxidative systems without excess drug loading and modification. Taken together, our findings provide new insights into the development of multifunctional molecular therapeutic agents for inflammatory osteolysis and other inflammatory diseases.

## Materials and methods

### Reagents and antibodies

Escherichia coli-derived lipopolysaccharide (LPS) was obtained from InvivoGen (San Diego, CA, USA). Recombinant mouse RANKL and macrophage colony-stimulating factor (M-CSF) were purchased from R&D (Minneapolis, MN, USA). Penicillin, streptomycin, and minimal essential medium alpha (α-MEM) were purchased from Gibco BRL (Gaithersburg, MD, USA). Fetal bovine serum (FBS) was purchased from Avantor (Radnor, PA, USA). The Cell Counting Kit-8 (CCK-8) was obtained from Beyotime Biotechnology (Shanghai, China). Methylisothiourea sulfate (SMT) was purchased from Selleck (Houston, TX, USA). Dihydroethidium (DHE) was purchased from Med Chem Express (Shanghai, China). The Prime Script RT reagent Kit and TB Green^TM^ Premix Ex Taq^TM^ II were obtained from Takara Biomedical Technology (Beijing, China). The primary antibody against iNOS (Thermofisher #PA1-036) was purchased from Thermofisher Scientific (Waltham, MA, USA). Primary antibodies against phospho-p65 (CST #3033), p65 (CST #8242), Keap1 (CST #4678), Nrf2 (CST #12721), HO-1 (CST #26416), and NQO1 (CST #62262) were purchased from Cell Signaling Technology (CST, Danvers, MA, USA). Primary antibodies against β-Actin (Affinity #AF7018), GAPDH (Affinity #AF7021), NFATc1 (Affinity #DF6446), CTSK (Affinity #DF6614), and TNF-α (Affinity #AF7014) were purchased from Affinity Biosciences (Cincinnati, OH, USA).

### General procedures

All syntheses were conducted under dry argon using Schlenk technique and vacuum-line system. All solvents were dried and distilled from appropriate drying agents prior to use. 1,2-Diamine-4-iodobenzene, di-tert-butyl dicarbonate ((BOC)_2_O) and other starting materials were commercially available without further purification. Bis(di-o-tolylphosphino-methyl)phenylphosphine (dTolmp) was prepared by the synthetic procedure described in the literature 31.

### Physical measurements

The ^1^H and ^31^P NMR spectra were performed on a Bruker Avance III (400 MHz) and ECZ600R (600 MHz) spectrometer with SiMe_4_ as the internal reference and H_3_PO_4_ as the external reference, respectively. Splitting patterns are designated as singlet (s), doublet (d), and triplet (t). Splitting patterns that could not be interpreted or easily visualized are designated as multiplet (m) and broad (br). The UV-Vis absorption spectra were measured on a Perkin-Elmer Lambda 35 UV-Vis spectrophotometer using a 10 mm path quartz cell. The high-resolution mass spectrometry (HRMS) was conducted on an Impact II mass spectrometer using dichloromethane and methanol mixtures as mobile phases. The emission and excitation spectra together with the emissive lifetimes were measured on Edinburgh FLS-920 fluorescence spectrometer. The emission spectra and lifetimes were measured upon excitation at 397 nm. The morphology of the material was observed by a field emission transmission electron microscope (FETEM, FEI Tecnai F20).

### Crystal structural determination

The X-ray single-crystal diffraction data of PtAu_2_-**1a** were collected on a Bruker D8 Venture diffractometer using IμS 3.0 microfocus source Mo-K*α* radiation (*λ* = 0.71073 Å) and PHOTON II CPAD detector. Frames were integrated with the Bruker SAINT software package (V8.38A) using a SAINT algorithm. Data were corrected for absorption effects using the multi-scan method (SADABS) 32. The structure was solved and refined using the Bruker SHELXTL Software Package, a computer program for automatic solution of crystal structures, and refined by the full-matrix least-squares method with ShelXle Version 4.8.6, a Qt graphical user interface for the SHELXL 33. All non-hydrogen atoms were refined anisotropically, whereas the hydrogen atoms were generated geometrically and refined using isotropic thermal parameters. CCDC 2211858 contains the supplementary crystallographic data for this paper. These data can be obtained free of charge from the Cambridge Crystallographic Data Centre via www.ccdc.cam.ac.uk/data_request/cif.

### Titration of PtAu_2_-1 cluster with NO

Sodium nitroprusside (Na_2_[Fe(CN)_5_NO]), a widely used exogenous NO donor 34, 35, was dissolved in distilled water as a 10 mM stock solution immediately before the experiments to avoid direct light exposure. The titration experiment was performed by addition of one- to eleven-fold aqueous solution of Na_2_[Fe(CN)_5_NO] to dimethyl sulfoxide (DMSO)-phosphate-buffered saline (PBS) (v/v = 1:1) solution of PtAu_2_-**1** cluster (the concentration of 1.0 × 10^-5^ M). The phosphorescent sensing behavior of PtAu_2_-**1** cluster to nitric oxide was monitored by the measurement of the emission spectrum change at a concentration of 1.0 × 10^-5^ M in DMSO-PBS (v/v = 1:1) solution upon excitation at 398 nm.

### Titration of PtAu_2_-1 cluster with L-cysteine

To investigate the possible binding interaction of PtAu_2_ cluster with cysteine, the titration experiment was performed by addition of one- to sixteen-fold aqueous solution of L-cysteine to a DMSO-PBS (v/v = 1:1) solution of PtAu_2_-**1** cluster (the concentration of 1.0 × 10^-5^ M). The binding interaction was monitored through the measurement of UV-Vis absorption and emission spectroscopy.

### Cellular uptake assay

RAW 264.7 murine macrophages were seeded in confocal dishes, then incubated with 100 ng/mL LPS plus 20 μg/mL PtAu_2_ clusters for 12 h. Then, cells were incubated with Hoechst 33342 (C1027; Beyotime Biotechnology, Shanghai, China) for nuclear staining. Meanwhile, a commercial kit for NO detection (DAF-FM DA) was used for comparison, according to the manufacturer's instructions. After several times of rinsing with warm PBS, the cells were observed in confocal laser scanning microscopy (CLSM).

For evaluation of lysosome colocalization, the cells were stained with Lyso-Tracker Red for another 30 min at 37 °C. After rinsing several times, the colocalization of PtAu_2_ clusters and endo/lysosomes was observed via CLSM.

### Quantitative real‑time PCR analysis

To analyze the mRNA level of pro-inflammatory genes, RAW 264.7 murine macrophages were cultured in 6-well plates at a density of 5 × 10^5^ cells/well, then stimulated with LPS (100 ng/mL) plus various concentrations of PtAu_2_ clusters (5, 10, and 20 μg/mL) for 24 h. To evaluate the mRNA level of OCs-related genes, bone marrow macrophages (BMMs) were cultured in 6-well plates at a density of 2 × 10^5^ cells/well supplemented with M-CSF (30 ng/mL) and RANKL (100 ng/mL), in the presence of various concentrations of PtAu_2_ clusters (5, 10, and 20 μg/mL) for 4 days. The total RNA of each group was extracted by using an Axygen RNA Miniprep Kit (Axygen, Union City, CA, USA), according to the manufacturer's instructions. Reverse transcription was performed based on RNA templates. TB Green^TM^ Premix Ex Taq^TM^ II was used for a real-time PCR (RT-qPCR) assay on an ABI 7500 Sequencing Detection System (Applied Biosystems, Foster City, CA). 5 μL of TB Green, 3 μL of ddH_2_O, 1 μL of cDNA, 0.4 μL of each primer and 0.2 μL ROX Dye2 were mixed to establish a 10 μL reaction system. Cycling conditions were set as 40 cycles (95 °C for 5 s and 60 °C for 30 s). The melting curves were checked to verify the amplification specificity. The relative gene expression was calculated using the comparative 2^-ΔΔCT^ method, as described previously 36. The housekeeping gene was set as *Gapdh*. The primer sequences are listed in Table S1.

### Immunofluorescence staining

RAW 264.7 murine macrophages were cultured with LPS (100 ng/mL) in the presence of various concentrations of PtAu_2_ clusters (5 and 20 μg/mL) for 24 h. Then, the cells were fixed, permeabilized, and blocked. After that, the cells were incubated with primary antibody against iNOS (1:100; PA1-036; Thermofisher, Waltham, MA, USA), p-p65 (1:100; CST #3033; CST, Danvers, MA, USA) and Nrf2 (1:100; CST #12721; CST, Danvers, MA, USA). The second antibodies for immunofluorescence were incubated for another 1 h. DAPI was used for nuclei staining. Then, the cells were detected via CLSM. The relative fluorescence intensity was analyzed by ImageJ software (NIH, Bethesda, MD, USA).

### Enzyme-linked immunosorbent assay (ELISA)

RAW 264.7 murine macrophages were cultured with LPS (100 ng/mL) in the presence of various concentrations of PtAu_2_ clusters (5, 10, and 20 μg/mL) for 24 h. Then the supernatants were collected, and the TNF-α, IL-1β, IL-6, and IL-10 contents of the supernatants were detected according to the ELISA kit instructions (Ruixin Biotech, Fujian, China).

### Detection of reactive oxygen species (ROS)

RAW 264.7 murine macrophages were seeded in confocal dishes, and cultured with 100 ng/mL LPS plus 20 μg/mL PtAu_2_ clusters for 24 h. After that, the cells were incubated with 5 μM DHE probe (HY-D0079; Med Chem Express, China) for 30 min at 37 °C. Hoechst 33342 was used for nuclear staining. After 3 times rinsing with warm PBS to remove the excess dye, the cells were detected via CLSM. ImageJ software (NIH, Bethesda, MD, USA) was used for semi-quantitative analysis of relative ROS fluorescence.

### GSH/GSSG measurement

GSH and GSSG Assay Kit (S0053; Beyotime Biotechnology, Shanghai, China) was used for the measurement of intracellular GSH/GSSG level, referred to the manufacturer's instructions. Briefly, RAW 264.7 murine macrophages were cultured in 6-well plates at a density of 5 × 10^5^ cells/well, then stimulated with LPS (100 ng/mL) plus various concentrations of PtAu_2_ clusters (5, 10, and 20 μg/mL) for 24 h. The cells were collected and lysed, then frozen and thawed in liquid nitrogen and 37 °C repetitively. After centrifugation, the supernatant of each sample was collected to measure total glutathione. The sample absorbance was measured at a wavelength of 412 nm, with the standard curve used for further calculation. The formula was shown as follow: GSH = Total Glutathione - GSSG × 2.

### Malonaldehyde (MDA) measurement

Cellular Lipid Peroxidation MDA Assay Kit (A003-4-1; Nanjing Jiancheng Bioengineering Institute, Jiangsu, China) was used for the measurement of intracellular MDA level, referred to the manufacturer's instructions. Briefly, RAW 264.7 murine macrophages were cultured in 6-well plates at a density of 5 × 10^5^ cells/well, then stimulated with LPS (100 ng/mL) plus various concentrations of PtAu_2_ clusters (5, 10, and 20 μg/mL) for 24 h. The cells were collected, lysed, and centrifuged at 12,000 g for 15 min. After that, the supernatant of each sample was collected for MDA measurement. The sample absorbance was measured at a wavelength of 532 nm.

### Superoxide Dismutase (SOD) measurement

Total Superoxide Dismutase Assay Kit with WST-8 (S0053; Beyotime Biotechnology, Shanghai, China) was used for the measurement of SOD activity. Briefly, RAW 264.7 murine macrophages were cultured in 6-well plates at a density of 5 × 10^5^ cells/well, then stimulated with LPS (100 ng/mL) plus various concentrations of PtAu_2_ clusters (5, 10, and 20 μg/mL) for 24 h. The cells were collected by 0.25% trypsin for subsequent experiments. The SOD activity was measured strictly according to the manufacturer's instructions. The results were presented as SOD activity relative to the control group, which was set at 100%.

### Tartrate-resistant acid phosphatase (TRAP) staining assay

BMMs were seeded into 96-well plates at a density of 8 × 10^3^ cells/well, then cultured with M-CSF (30 ng/mL) and RANKL (100 ng/mL), in the presence of various concentrations of PtAu_2_ clusters (2.5, 5, and 10 μg/mL). The culture medium was replaced every 2 days until the formation of OCs was observed on day 5. After a 20 min fixation with 4% paraformaldehyde (PFA), the cells were stained with TRAP staining solution at 37 °C for 1 h. OCs were defined as TRAP-positive cells with more than three nuclei. An optical microscope (Olympus, Tokyo, Japan) was used for image capturing. The OCs number and area were quantified by ImageJ software (NIH, Bethesda, MD, USA).

### Detection of podosome actin belt

BMMs were cultured with M-CSF (30 ng/mL) and RANKL (100 ng/mL), in the presence of various concentrations of PtAu_2_ clusters (2.5, 5, and 10 μg/mL). After mature OC formation, the cells were fixed and permeabilized, then incubated with TRITC Phalloidin (1:100; CA1610; Solarbio, Beijing, China) for 40 min. After that, the nuclei were stained with DAPI for 5 min. The cells were washed with PBS three times, then observed via CLSM. The number of podosome actin belts was quantified by ImageJ software (NIH, Bethesda, MD, USA).

### Bone resorption assay

At a density of 1 × 10^4^ cells/well, BMMs were seeded into 96-well Corning Osteo Assay Surface plates (Corning, NY, USA), then cultured with M-CSF (30 ng/mL) and RANKL (100 ng/mL), in the presence of various concentrations of PtAu_2_ clusters. The culture medium was replaced every 2 days until the formation of mature OCs. After culture for another 1 day, the mature OCs were eliminated by 5% sodium hypochlorite. The resorption pits were imaged using an optical microscope. The bone resorption area was analyzed by ImageJ software (NIH, Bethesda, MD, USA).

### RNA sequencing

RNA sequencing was performed to explore the potential mechanisms for the anti-inflammatory effect of PtAu_2_ clusters. Briefly, RAW 264.7 murine macrophages were seeded in 6-well plates at a density of 5 × 10^5^ cells/well, then cultured with 100 ng/mL LPS plus 20 μg/mL PtAu_2_ clusters for 24 h. TRIzol reagent was used to collect the total RNA. The gene expression level was measured by Biomarker Technologies (Beijing, China). Gene ontology (GO) enrichment analysis and Kyoto Encyclopedia of Genes and Genomes (KEGG) pathway enrichment analysis were performed using BMKCloud (www.biocloud.net).

### Immunoblotting

For RAW 264.7 murine macrophages, the cells were seeded in 6-well plates at a density of 5 × 10^5^ cells/well, then stimulated with 100 ng/mL LPS plus various concentrations of PtAu_2_ clusters (5, 10, and 20 μg/mL) for 24 h. For BMMs, the cells were seeded in 6-well plates at a density of 3 × 10^5^ cells/well with M-CSF (30 ng/mL) and RANKL (100 ng/mL), then treated in the presence or absence of 10 μg/mL PtAu_2_ clusters for 0, 1, 3 and 5 days. Then, the total protein of each sample was extracted by using whole cell lysis buffer with a protease inhibitor cocktail (P8340; Sigma-Aldrich, Shanghai, China). After the lysates were centrifuged at 12,000 g for 15 min, the supernatant of each sample was collected. Nuclear proteins were extracted via the nuclear extraction kit (Thermofisher, Waltham, MA, USA) by high-speed centrifugation, according to the manufacturer's instructions. Protein concentrations were measured using Bicinchoninic acid (BCA) assay (P0012; Beyotime Biotechnology, Shanghai, China). Then, the proteins were dissolved in the loading buffer, separated by 10% SDS-PAGE, and transferred to 0.22 µm polyvinylidene fluoride (PVDF) membranes. After 1 h blocking in 5% BSA in 1 × TBST (Tris-buffered saline with Tween 20) at room temperature, the membranes were incubated with the primary antibodies (β-Actin, 1:1000; GAPDH, 1:1000; iNOS, 1:1000; p-p65, 1:1000; p65, 1:1000; Keap1, 1:1000; Nrf2, 1:500; HO-1, 1:1000; NQO1, 1:1000) overnight at 4 ºC. After that, the secondary antibodies were incubated with membranes for 1 h at room temperature. Odyssey V3.0 image scanning (Li-COR. Inc., Lincoln, NE, USA) was used for blot detection. The blot results were analyzed and quantified by ImageJ software (NIH, Bethesda, MD, USA).

### Immunoprecipitation

For each sample, a total of 600 μg protein lysates were pre-incubated with IgA/G beads (Sigma, Shanghai, China). Anti-Keap1 antibody (Keap1, 1:50) and protein IgA/G beads were used to precipitate with endogenous Keap1. Then, the Keap1-Nrf2 immuno-complex was subjected to Western blotting.

### LPS-induced calvarial osteolysis model

The Animal Care and Experiment Committee of Ninth People's Hospital Affiliated to Shanghai Jiao Tong University School of Medicine (SH9H-2022-A874-1) approved the animal protocol. All experiments were performed according to the guidelines for Ethical Conduct in the Care and Use of Nonhuman Animals in Research by the American Psychological Association. The murine calvarial osteolysis model was used to evaluate the *in vivo* therapeutic effect of PtAu_2_ clusters on inflammatory response and bone resorption, based on the previous reports 37, 38. Briefly, fifteen 8-week-old C57/BL6 male mice (approximate weight 25 ± 2 g) were randomly divided into three groups with five animals per group: (1) Sham group (with PBS injection); (2) LPS group (10 mg/kg body weight of LPS and PBS injection); (3) PtAu_2_ group (10 mg/kg body weight of LPS and 20 μg/mL PtAu_2_ clusters injection). Collagen sponges (4 mm × 4 mm × 2 mm) soaked with PBS or LPS were implanted in the middle of the calvaria under general anesthesia. 3 days after implantation, all the mice were subject to subcutaneous injections of calvarias every other day over another 11-day period. After experiments, the mice were euthanized. The whole calvaria was harvested and washed with PBS, then fixed in 4% PFA for 48 h for radiographic and histological analysis.

### Micro-computed tomography

Micro-computed tomography (CT) scanning was performed using a high-resolution micro-CT (μCT-100, SCANCO Medical AG, Switzerland). The resolution of the scanning was 10 μm; the X-ray energy was set at 70 kv, 200 μA; and a fixed exposure time was 300 ms. The microstructure indicators of bone volume/tissue volume (BV/TV), trabecular number (Tb.N), trabecular separation (Tb.Sp), and trabecular number (Tb.Th) were measured in a three-dimensional region of interest (ROI) using evaluation analysis software (Version: 6.5-3, SCANCO Medical AG, Switzerland).

### Histological and immunohistochemical analysis

After micro-CT scanning, the samples were decalcified in 10% EDTA (pH = 7.4) for 4 weeks, then embedded in paraffin. Histological sections were prepared for hematoxylin and eosin (H&E) and TRAP staining. Immunofluorescence staining was performed with antibodies against TNF-α (1:100; AF7014; Affinity, Cincinnati, OH, USA), Nrf2 (1:100; AF0639; Affinity, Cincinnati, OH, USA), and HO-1 (1:100; AF5393; Affinity, Cincinnati, OH, USA). The images of stained slices were captured under a high-quality microscope (Leica DM4000B). The relative fluorescence of stained slices was quantified by ImageJ software (NIH, Bethesda, MD, USA). The number of OCs and TRAP-positive multinucleated OCs per field (Oc.S/BS) were calculated.

### *In vivo* biosafety evaluation

14 days after model establishment, the major organs of mice were collected for H&E staining. The systemic toxicity of PtAu_2_ clusters was evaluated based on the H&E staining of the heart, liver, spleen, lung, and kidney. Meanwhile, blood routine examinations and serum biochemical analyses were performed by collecting the blood samples.

### *In vivo* biodistribution of PtAu_2_ clusters

To detect the biodistribution and metabolism of PtAu_2_ clusters in major organs, 8-week-old C57/BL6 male mice (n = 3) were subcutaneously injected with PtAu_2_ clusters on calvarias at a dose of 100 μg/mL (100 μL). Two days after injection, mice were sacrificed and the major organs were harvested. Then, the organ tissues were weighed, homogenized, and dissolved in aqua regia. Inductively coupled plasma mass spectrometry (ICP-MS) was used to measure the amounts of Au and Pt in each sample, and the percentage of injected dose per gram of tissue (%ID g^-1^) was calculated.

### Statistical Analysis

The results were analyzed by using the Prism 8.0 statistical software package (GraphPad Software Inc, San Diego, CA, USA). The data were uniformly presented in the form of mean ± standard deviation (SD). Differences between the two groups were analyzed using the two-tailed unpaired Student's t-test, after the homogeneity test of variance. One-way analysis of variance (ANOVA) with Tukey's post hoc tests was used for multiple group comparisons. Significant differences were determined to be at *p < 0.05 and **p < 0.01.

## Results and Discussion

### Synthesis and characterization of PtAu_2_ clusters

**Figure 2:**
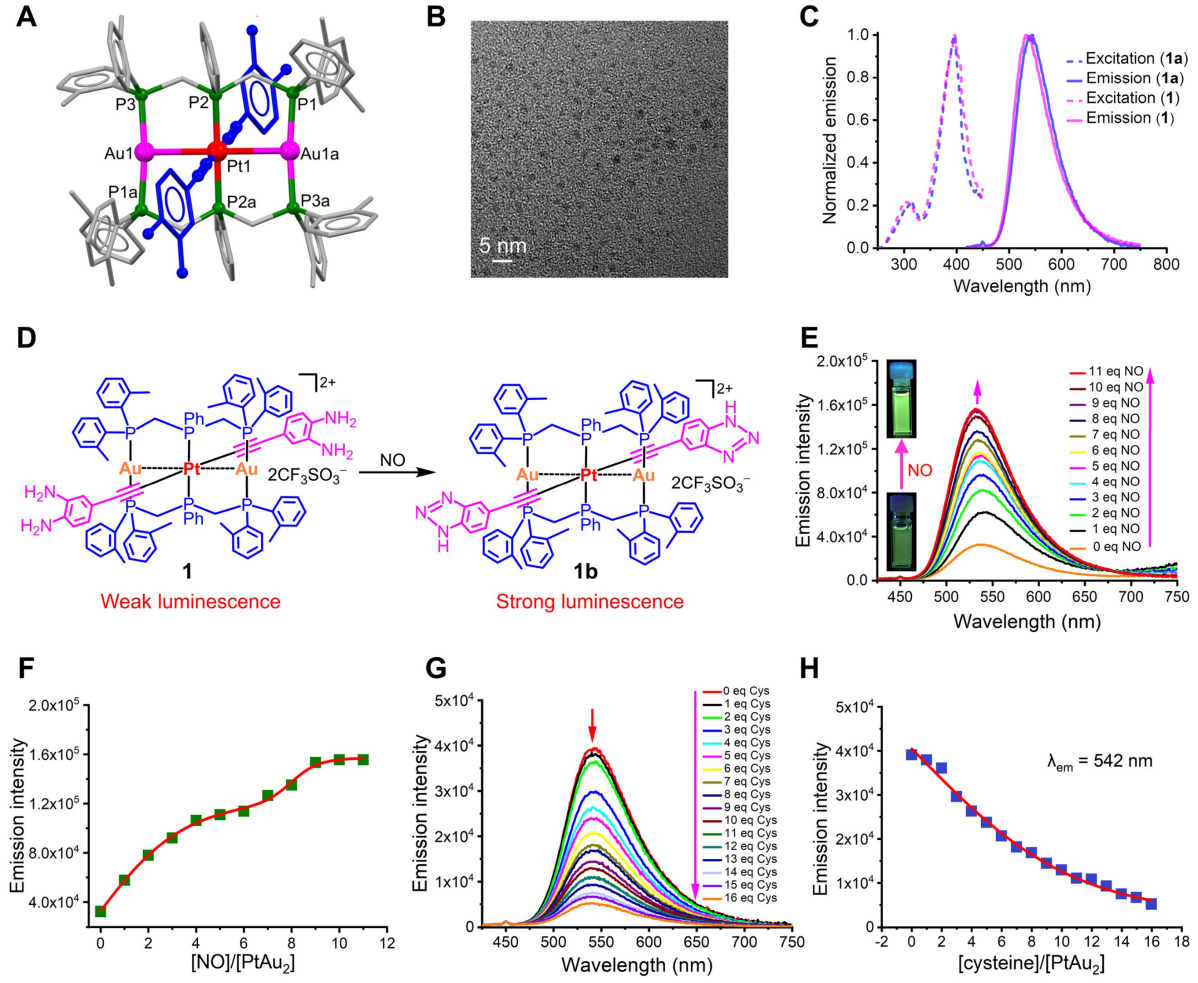
** Characterization of PtAu_2_ clusters. (A)** Coordination structure of the PtAu_2_-**1** cluster. **(B)** TEM image of the PtAu_2_-**1** clusters. **(C)** Excitation and emission spectra of the BOC-protected PtAu_2_-**1a** and the BOC-removed PtAu_2_-**1** clusters at a concentration of 1.0 × 10^-5^ M in DMSO-PBS (v/v = 1:1). **(D)** Schematic diagram showing the phosphorescent turn-on effect of the PtAu_2_-**1** cluster in response to NO. **(E)** NO-responsive emission spectra change of the PtAu_2_-**1** cluster at a concentration of 1.0 × 10^-5^ M in DMSO-PBS (v/v = 1:1) solution, showing a gradual increase in emission intensity with the addition of 1 to 11 equivalent Na_2_[Fe(CN)_5_NO]. **(F)** Dependence of phosphorescence intensity on the molar ratio of Na_2_[Fe(CN)_5_NO] *vs* PtAu_2_-**1** cluster. **(G)** Emission spectral change of the PtAu_2_-**1** cluster (1.0 × 10^-5^ M) in DMSO-PBS (v/v = 1:1) solution in response to an aqueous solution of L-cysteine, showing a gradual decrease in the peak at 542 nm. **(H)** Dependence of emission intensity at 542 nm on the molar ratio of L-cysteine* vs* PtAu_2_-**1** cluster.

Because 4-ethynylbenzene-1,2-diamine is highly sensitive to air, PtAu_2_ clusters were first synthesized using t-butyloxy carbonyl (BOC) protected ethynyl ligands. As shown in Figure 1**A** and Scheme S1, the BOC-protected PtAu_2_-**1a** cluster was prepared by reacting the mononuclear Pt(II) precursors, bis(di-o-tolylphosphino-methyl)phenylphosphine (dTolmp), chloro(tetrahydrothiophene)gold(I) (Au(tht)Cl), and NaSO_3_CF_3_ in a 1:2:2:2 molar ratio at room temperature 39, 40. The structure of the BOC-protected PtAu_2_-**1a** cluster (Table S2) was determined using single crystal X-ray diffraction, which confirmed that the PtAu_2_ framework was supported by double dTolmp ligands. The PtAu_2_ cluster (Figure S1) is highly stabilized by four five-membered chelating rings, as well as significant d^8^-d^10^ heterometallic interactions as the Pt-Au distance (2.9245(2) Å) is much shorter than the sum of the van der Waals radii for Pt and Au (ca. 3.3 Å). Removal of the BOC protective groups gave the target PtAu_2_-**1** cluster in 45% yield (Figure 2**A**). Both the PtAu_2_-**1** and BOC-protected PtAu_2_-**1a** clusters were fully characterized using ^1^H and ^31^P NMR spectrometry (Figures S2, S3, S5, and S6) and high-resolution mass spectrometry (Figures S4 and S7). Transmission electron microscopy (TEM) further indicated that the PtAu_2_-**1** clusters were ultrasmall and widely dispersed, with a uniform nanomorphology and a diameter of approximately 2 nm (Figure 2**B**).

Upon excitation at λ_ex_ = 398 nm, the BOC-protected PtAu_2_-**1a** cluster displayed intense green luminescence, peaking at 534 nm with an emissive lifetime of 2.0 μs and 26% quantum yield in DMSO-PBS (v/v = 1:1) at room temperature (Figure 2**C**). The long-lived emission with a micro-second range lifetime and a large Stokes shift suggested that phosphorescent characteristics may arise from the mixed triplet excited states of ^3^[acetylide → dTolmp] ^3^LLCT (ligand-to-ligand charge transfer) and ^3^[acetylide → PtAu_2_] ^3^LMCT (ligand-to-metal charge transfer) transitions 39, 40. In contrast, the PtAu_2_-**1** cluster exhibited relatively weak luminescence centered at 542 nm with an emissive lifetime of 1.7 μs and a quantum yield of 1.9%. The much weaker phosphorescence of the PtAu_2_-**1** cluster compared to the BOC-protected PtAu_2_-**1a** cluster is likely due to the strong electron-donating character of the diamino groups, which facilitate intramolecular electron transfer that deactivates triplet excited states through non-radiative decay.

The long-term stability of the PtAu_2_-**1** cluster was further characterized using UV-Vis absorption and phosphorescence spectra. The inappreciable changes in the bands centered at 395 and 495 nm due to [acetylide → dTolmp] LLCT (ligand-to-ligand charge transfer) and [acetylide → PtAu_2_] ligand-to-metal charge transfer (LMCT) transitions, imply that the PtAu_2_-**1** cluster maintains sufficient stability in the DMSO-PBS (v/v = 1:1) solution after it has been put aside for three days (Figure S8). Likewise, the inappreciable changes in the phosphorescent bands centered at 534 nm, ascribed to ^3^[acetylide → dTolmp] LLCT (ligand-to-ligand charge transfer) and ^3^[acetylide → PtAu_2_] LMCT triplet excited states, suggest that the PtAu_2_-**1** cluster is sufficiently stable in DMSO-PBS (v/v = 1:1) solution after it has been put aside for three days (Figure S9).

The binding of weakly phosphorescent PtAu_2_-**1** cluster to molecules, such as NO, through diamino groups to produce a triazole ring effectively inhibited intramolecular electron transfer 41, resulting in dramatically enhanced phosphorescence (Figure 2**D**). Therefore, we performed phosphorescent sensing experiments by titrating the PtAu_2_-**1** cluster in DMSO-PBS (v/v = 1:1) with an aqueous solution of sodium nitroprusside (Na_2_[Fe(CN)_5_NO]), a widely used exogenous NO donor 42, 43. As depicted in Figures 2**E** and** 2F**, the addition of sodium nitroprusside progressively intensified the phosphorescent emission peak at 542 nm, resulting in a four-fold phosphorescent enhancement with 10 equivalents of sodium nitroprusside. In addition, gradual titration with sodium nitroprusside caused a progressive blue-shift of the emission peak for the PtAu_2_-**1** cluster to 533 nm, due to the formation of PtAu_2_-**1b** cluster (Figure 2**E**) with the benzotriazole-acetylide ligand. Thus, the PtAu_2_-**1** cluster displays excellent phosphorescent turn-on effects in response to NO and is a promising biosensor for detecting NO in inflammatory environments.

As an effective binding agent for cysteine residues, Au^+^ has been reported to react with cysteine thiol groups in specific peptides and proteins, thereby exerting biological effects 44, 45. For instance, Au^+^ can exert anti-inflammatory effects by inhibiting inflammatory ligand-receptor recognition and increasing the release of protective proteins. To further investigate the possible binding interactions between the heterometallic PtAu_2_ clusters and cysteine, titration experiments were performed by adding an aqueous L-cysteine solution to the PtAu_2_-**1** cluster in DMSO-PBS (v/v = 1:1). As shown in Figures S10 and S11, the 390 nm absorbance peak of the PtAu_2_-**1** cluster was dose-dependently reduced by the addition of cysteine, which also progressively reduced its emission intensity at 542 nm (Figures 2**G** and 2**H**). Taken together, these results suggest a potential binding interaction between cysteine and the PtAu_2_-**1** cluster, which may provide this heterometallic cluster with anti-inflammatory activity.

### Biocompatibility and cellular uptake of PtAu_2_ clusters

**Figure 3:**
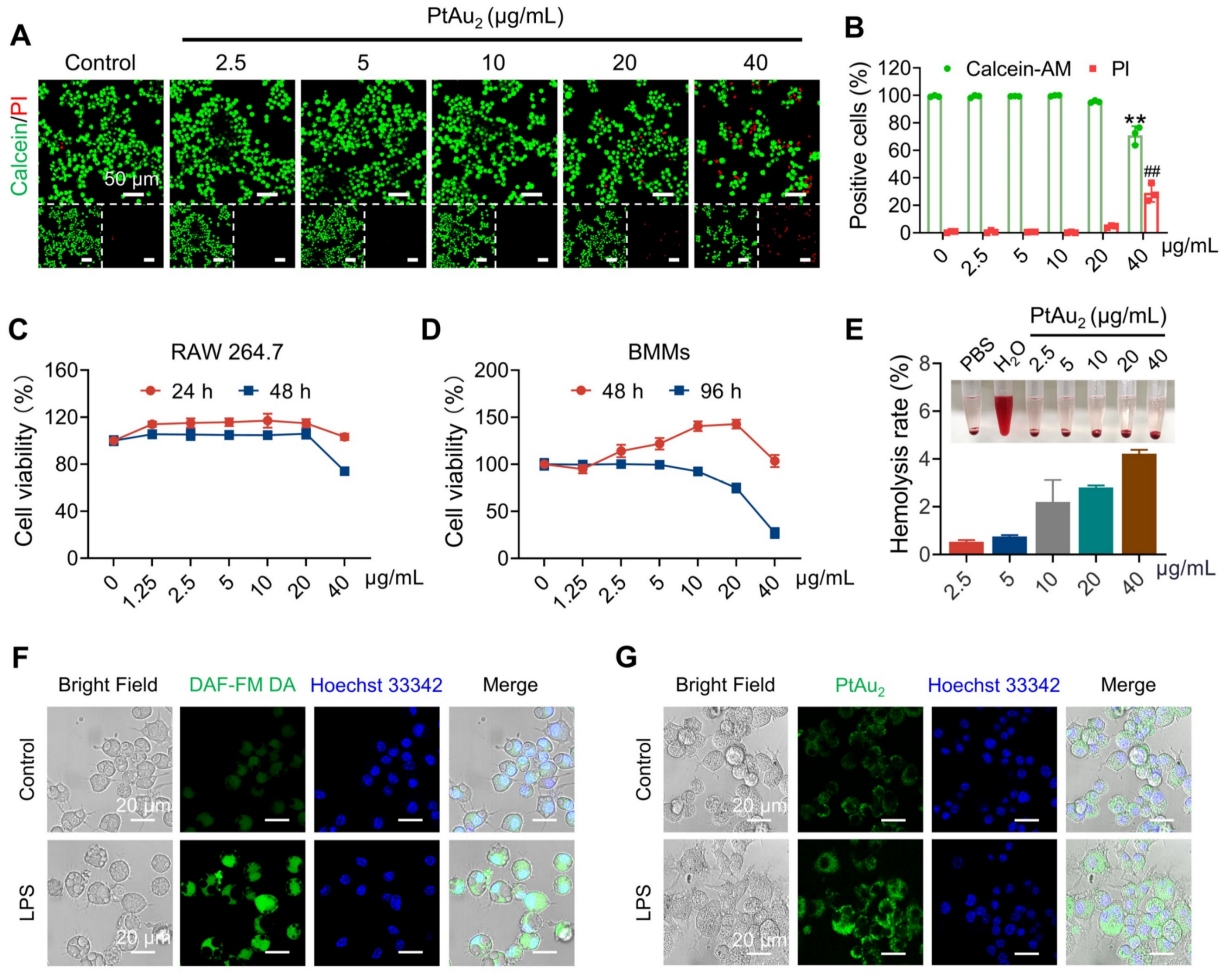
** Biocompatibility and cellular uptake of PtAu_2_ clusters. (A)** Live/dead staining of RAW 264.7 macrophages treated with various concentrations of PtAu_2_ clusters for 24 h. **(B)** Quantitative analysis of Calcein-AM positive cells and PI positive cells. **(C)** Viability of RAW 264.7 macrophages treated with various concentrations of PtAu_2_ clusters for 24 and 48 h. **(D)** Viability of murine BMMs treated with various concentrations of PtAu_2_ clusters for 48 and 96 h. **(E)** Hemolysis assay of PtAu_2_ clusters. **(F)** Representative images of LPS-activated RAW 264.7 macrophages stained with DAF-FM DA and detected by CLSM. **(G)** Representative images of LPS-activated RAW 264.7 macrophages incubated with PtAu_2_ clusters detected by CLSM. Data represent the mean ± SD of three independent experiments (two-tailed unpaired Student's t-test). ** and ^##^ *p* < 0.01 compared to the 0 μg/mL group.

Next, we investigated the biocompatibility and cellular uptake behavior of PtAu_2_ clusters. Live/dead staining was performed to evaluate the cytotoxicity of PtAu_2_ clusters at various concentrations. As shown in Figures 3**A** and 3**B**, PtAu_2_ clusters exhibited neglectable cytotoxicity to RAW 264.7 macrophages at a dose of 20 μg/mL. However, an increased number of PI-positive cells and a reduced number of Calcein-AM-positive cells were observed when the concentration reached 40 μg/mL. To further evaluate the effect of PtAu_2_ clusters on cell viability, CCK-8 assays were performed. As shown in Figure 3**C**, PtAu_2_ clusters hardly affected the viability of RAW 264.7 macrophages, even at a dose of 40 μg/mL for 24 h. However, clear cytotoxicity was observed at a concentration of 40 μg/mL after prolonged treatment for 48 h. Similarly, PtAu_2_ clusters displayed negligible cytotoxicity in BMMs at a dose of 40 μg/mL after 48 h (Figure 3**D**), but dose-dependently reduced cell viability was observed at concentrations of 20 μg/mL or higher when treated for 96 h. Consistently, flow cytometry revealed that PtAu_2_ clusters only marginally affected cell cycle phases at doses of up to 20 μg/mL (Figure S12). To further explore whether PtAu_2_ clusters adversely affected blood cell homeostasis, hemolysis tests were performed by incubating red blood cells (RBCs) with various concentrations of PtAu_2_ clusters. As shown in Figure 3**E**, PtAu_2_ clusters slightly reduced RBC viability at doses up to 20 μg/mL, with a hemolysis rate lower than 3%. Collectively, these results demonstrate that PtAu_2_ clusters exhibit satisfactory biocompatibility at concentrations up to 20 μg/mL in RAW 264.7 macrophages and could be used at concentrations of 10 μg/mL or below in BMMs without compromising cell viability.

Having evaluated their biocompatibility, we examined the cellular uptake behavior of PtAu_2_ clusters and the feasibility of NO-responsive phosphorescence in inflammatory environments. Since NO is an inflammatory molecule produced by inflammatory macrophages, we detected NO-responsive phosphorescence in RAW 264.7 macrophages stimulated by LPS for 12 h and treated with PtAu_2_ clusters at a concentration of 20 μg/mL, and used a commercial NO detection (DAF-FM DA) kit for comparison. As shown in Figure 3**F**, the fluorescence intensity of DAF-FM DA was markedly enhanced after LPS stimulation, indicating an increase in NO production. Similarly, the phosphorescence intensity of PtAu_2_ clusters was significantly enhanced in LPS activated-macrophages but not in unstimulated macrophages (Figures 3**G** and S13). Weak phosphorescence was observed in LPS-activated macrophages following the addition of SMT, an iNOS inhibitor, suggesting that phosphorescence enhancement was mainly triggered by NO generation (Figure S14). Furthermore, we observed that most PtAu_2_ clusters escaped lysosomes after 12 h, with little colocalization observed due to the cationic property of the PtAu_2_ clusters (Figure S15). Taken together, these results demonstrate that PtAu_2_ clusters exhibit satisfactory cellular uptake and NO-responsive phosphorescence enhancement in inflammatory macrophages.

### Anti-inflammatory effects of PtAu_2_ clusters *in vitro*

**Figure 4:**
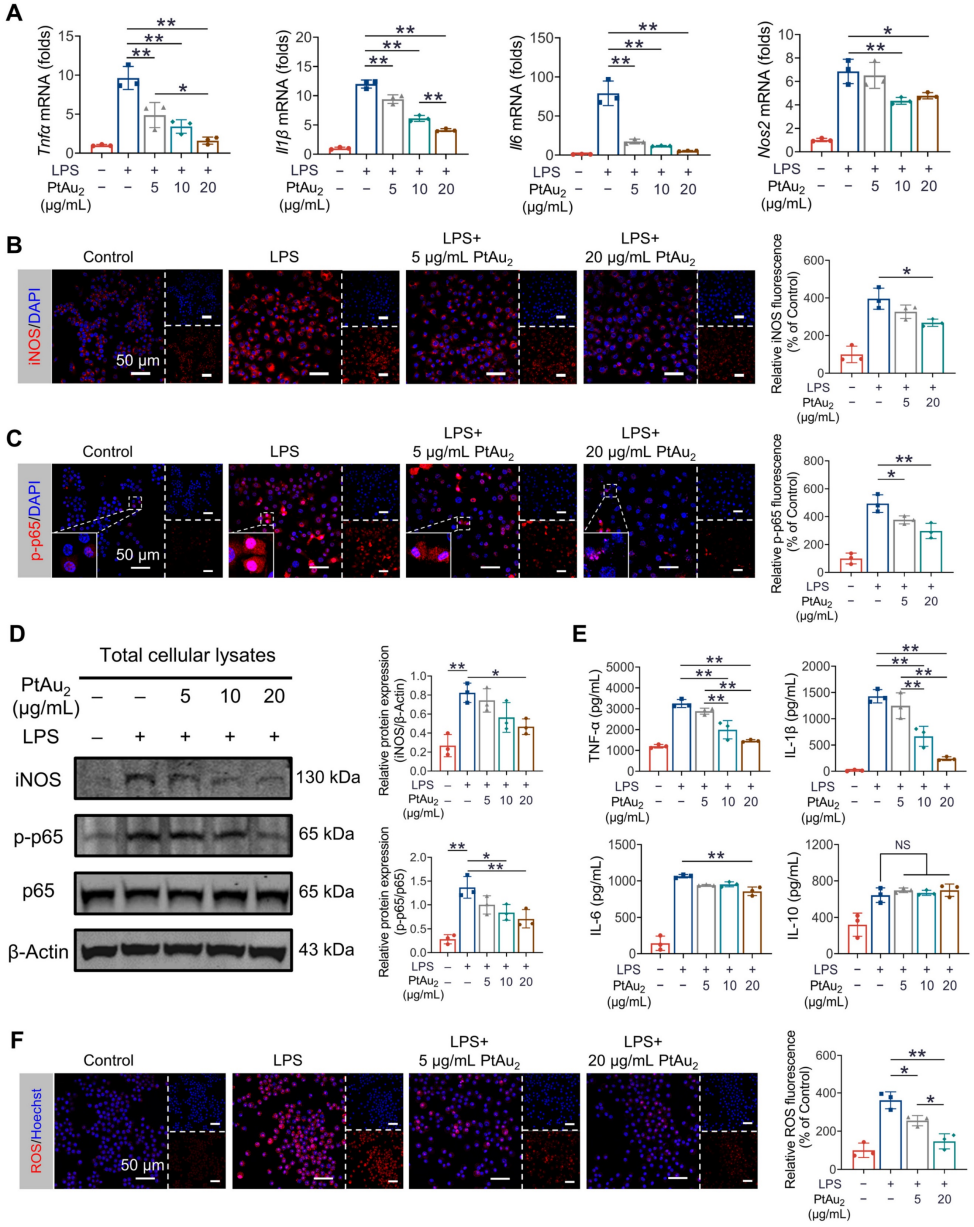
PtAu_2_ clusters suppress inflammatory reactions in LPS-stimulated RAW 264.7 macrophages. (A) RT-qPCR analysis of pro-inflammatory gene expression in LPS-activated RAW 264.7 macrophages treated with various concentrations of PtAu_2_ clusters. (B) iNOS immunofluorescence in LPS-activated RAW 264.7 macrophages treated with various concentrations of PtAu_2_ clusters. (C) p-p65 immunofluorescence in LPS-activated RAW 264.7 macrophages treated with various concentrations of PtAu_2_ clusters. (D) Expression of iNOS, p-p65, and p65 in total cellular lysates after treatment with various concentrations of PtAu2 clusters. (E) ELISA results for inflammatory cytokines production by LPS-activated RAW 264.7 macrophages treated with various concentrations of PtAu_2_ clusters. (F) Confocal images of LPS-activated RAW 264.7 macrophages treated with various concentrations of PtAu_2_ clusters and stained with DHE. Data represent the mean ± SD of three independent experiments (one-way ANOVA with Tukey's post hoc test). *p < 0.05; **p < 0.01.

Encouraged by the satisfactory biocompatibility and cellular uptake behavior of PtAu_2_ clusters, we next explored whether these nanoclusters exhibited anti-inflammatory effects in inflammatory macrophages. RAW 264.7 macrophages were challenged with LPS in the presence of various concentrations of PtAu_2_ clusters (5, 10, and 20 μg/mL) for 24 h. As shown in Figure 4**A**, RT-qPCR assays revealed that *Tnfα*, *Il1β*, *Il6*, and *Nos2* mRNA expression was upregulated following LPS stimulation but was dose-dependently inhibited after treatment with PtAu_2_ clusters. Next, we investigated the effect of PtAu_2_ clusters precursors on the inflammatory reaction by RT-qPCR. The results demonstrated that, unlike the complete PtAu_2_ clusters, the precursors of PtAu_2_ clusters (dTolmp, Pt(PPh_3_)_2_ and Au(tht)Cl) exhibited a marginal inhibitory effect on pro-inflammatory gene expression (Figure S16), indicating that the structural integrity is important for the anti-inflammatory effect of PtAu_2_ clusters. Consistently, immunofluorescence staining revealed that iNOS expression was significantly elevated following LPS stimulation, while it was suppressed by various concentrations of PtAu_2_ clusters in LPS-activated RAW 264.7 macrophages (Figure 4**B**). LPS stimulation also markedly upregulated the phosphorylation and nuclear translocation of NF-κB p65, which were dose-dependently inhibited by PtAu_2_ clusters (Figure 4**C**). Furthermore, we investigated the expression of iNOS, p-p65, and p65 using western blotting. As shown in Figure 4**D**, the expression of iNOS and the phosphorylation of p65 were markedly upgraded following LPS stimulation, whereas they significantly decreased by treatment with PtAu_2_ clusters in a dose-dependent manner. In addition, to determine the effect of PtAu_2_ clusters on inflammatory cytokines at the protein level, we performed ELISA to measure the production of inflammatory cytokines. PtAu_2_ clusters dose-dependently reduced the production of the pro-inflammatory cytokines TNF-α, IL-1β, and IL-6 but hardly affected the production of the anti-inflammatory cytokine IL-10 (Figure 4**E**). Taken together, these results suggest that PtAu_2_ clusters suppress inflammatory reactions in LPS-stimulated RAW 264.7 macrophages.

When activated by various inflammatory stimuli, macrophages exhibit respiratory bursts and produce excessive ROS, which leads to oxidative stress. Oxidative stress may further aggravate inflammatory reactions, triggering a vicious cycle 46, 47. Therefore, we investigated the effect of PtAu_2_ clusters on ROS overproduction in inflammatory macrophages. As shown in Figure 4**F**, PtAu_2_ clusters effectively reduced intracellular ROS levels, with weaker DHE fluorescence than that of the LPS-activated group. Moreover, the level of MDA, a well-established marker of oxidative stress, was efficiently reduced by PtAu_2_ clusters treatment (Figure S17). Furthermore, both the intracellular GSH level and SOD activity were restored by treatment with PtAu_2_ clusters in a dose-dependent manner (Figure S18 and S19), thereby increasing the cellular resistance to oxidative stress. Collectively, these data suggest that PtAu_2_ clusters suppress oxidative stress and increase the anti-oxidative capacity of LPS-stimulated RAW 264.7 macrophages.

### Effect of PtAu_2_ clusters on OC formation, fusion, and function

**Figure 5:**
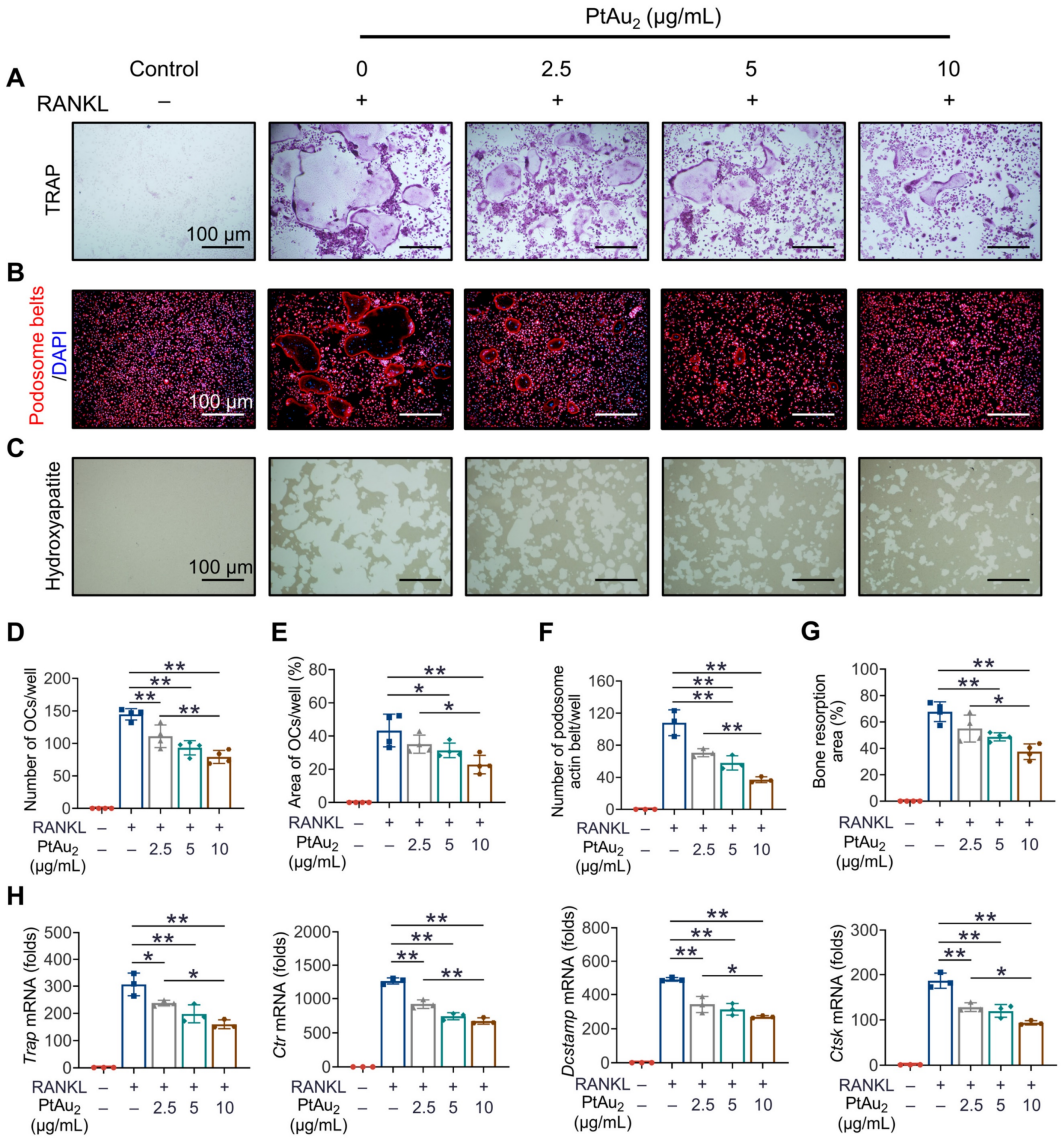
** PtAu_2_ clusters reduce RANKL-induced osteoclastogenesis, podosome actin belt formation, and OC-mediated bone resorption. (A)** Representative tartrate-resistant acid phosphatase (TRAP) staining images of BMMs stimulated with RANKL for 5 days and treated with various concentrations of PtAu_2_ clusters. **(B)** Representative immunofluorescence images of podosome actin belts in BMMs stimulated with RANKL for 5 days and treated with various concentrations of PtAu_2_ clusters. **(C)** Representative images of bone resorption pits on Osteo Assay Surface plates coated with hydroxyapatite produced by OCs treated with various concentrations of PtAu_2_ clusters. **(D)** Number of OCs per well. **(E)** Area of OCs per well. **(F)** The number of podosome actin belts per well. **(G)** Bone resorption area per well. **(H)** RT-qPCR results of OCs-related gene expression in BMMs cultured with RANKL for 4 days in presence of various concentrations of PtAu_2_ clusters. **(D, E, and G)** Data represent the mean ± SD of four independent experiments (one-way ANOVA with Tukey's post hoc test). **(F and H)** Data represent the mean ± SD of three independent experiments (one-way ANOVA with Tukey's post hoc test). *p < 0.05; **p < 0.01.

Having demonstrated the potent anti-inflammatory effects of PtAu_2_ clusters, we next investigated whether PtAu_2_ clusters exert inhibitory effects against OCs, the principal effector cells of inflammatory osteolysis. A maximum concentration of 10 μg/mL of PtAu_2_ clusters was used based on their cytotoxicity in BMMs. As shown in Figure 5**A**, BMMs differentiated into mature OCs with multiple nuclei in the presence of RANKL for five days. However, the formation of TRAP-positive OCs was reduced in a dose-dependent manner by various concentrations of PtAu_2_ clusters (2.5, 5, and 10 μg/mL), which reduced the number and area of the OCs (Figure 5**D** and 5**E**). Notably, the nuclear number of mature OCs also significantly decreased in a dose-dependent manner following treatment with PtAu_2_ clusters, indicating the inhibitory effect of PtAu_2_ clusters on OC fusion (Figure S20). To further evaluate the effect of PtAu_2_ clusters on OC fusion, cytoskeletal podosome actin belts, which indicate OC precursor cell fusion ability, were visualized by immunofluorescence. As shown in Figures 5**B** and 5**F**, PtAu_2_ clusters dose-dependently suppressed the formation of podosome actin belts, further indicating that PtAu_2_ clusters reduced OC fusion. In addition, we used Osteo Assay Surface plates with hydroxyapatite coating to evaluate the effect of PtAu_2_ clusters on bone resorption, an important function of mature OCs. Extensive bone resorption was observed when BMMs were stimulated with RANKL for 6 days; however, the bone resorption area was reduced in a dose-dependent manner by various concentrations of PtAu_2_ clusters (Figures 5**C** and 5**G**). To further investigate the effect of PtAu_2_ clusters on transcript levels in OCs, RT-qPCR was performed to analyze the expression of OC-related genes, including *Trap* (osteoclast differentiation), *Ctr* (osteoclast differentiation), *Dcstamp* (osteoclast fusion), and *Ctsk* (bone resorption). As expected, PtAu_2_ clusters markedly downregulated the expression of OC-related genes in a dose-dependent manner (Figure 5**H**). Moreover, western blotting results demonstrated that the expression of NFATc1 (a key transcriptional factor in osteoclastogenesis) and CTSK (a key protein in OCs-mediated bone resorption) gradually increased over time in the control group but was reduced after treatment with 10 μg/mL PtAu_2_ clusters (Figure S21). Taken together, these results indicate that PtAu_2_ clusters effectively reduce RANKL-induced OC formation, fusion, and function in BMMs.

### Anti-inflammatory mechanisms of PtAu_2_ clusters

**Figure 6:**
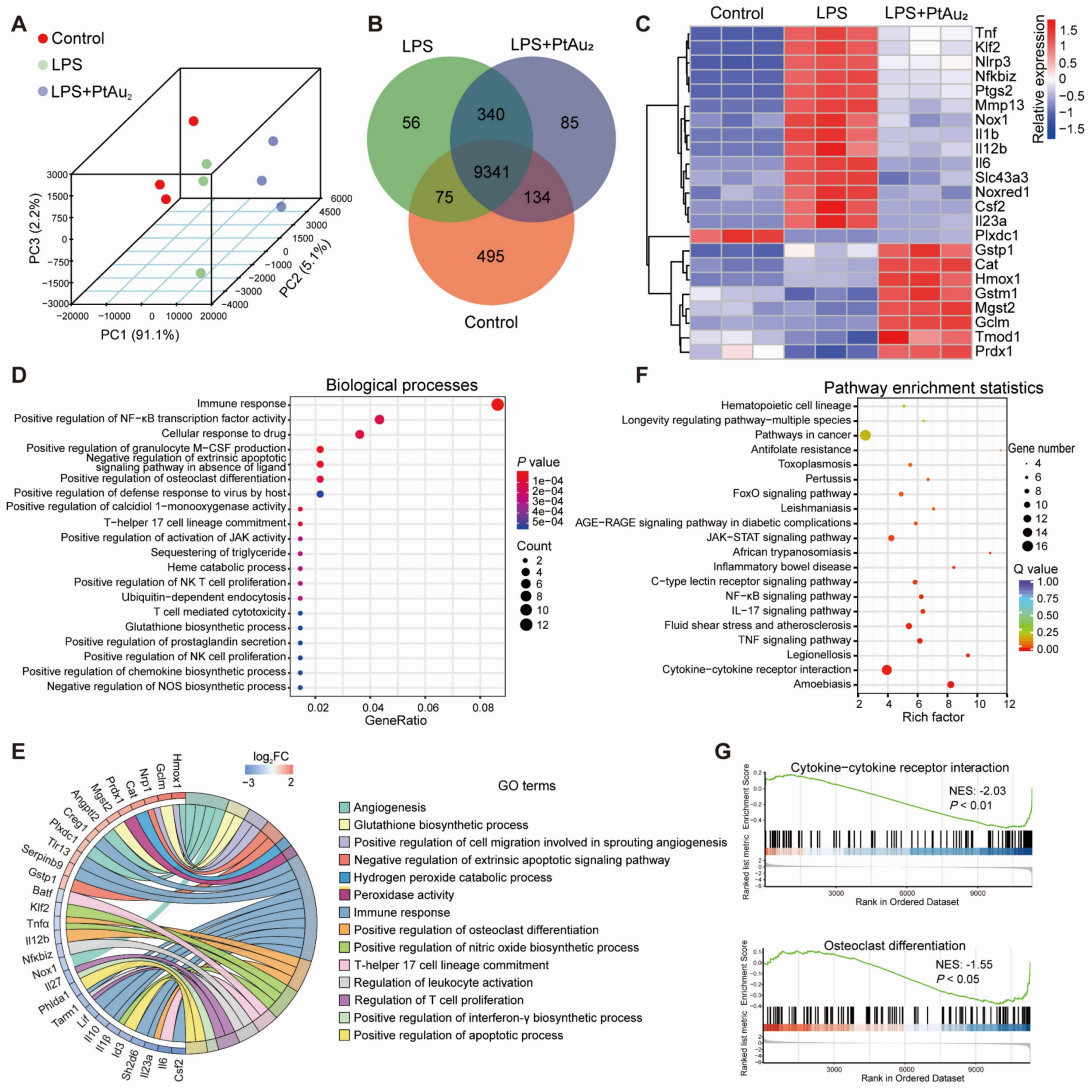
** Transcriptomic analysis of the anti-inflammatory mechanisms of PtAu_2_ clusters. (A)** PCA of the transcriptomic profiles in the Control, LPS, and LPS + PtAu_2_ groups. **(B)** Venn diagram. **(C)** Clustered heat map of the representative inflammation-related genes (fold change ≥ 2.0 and *p* < 0.05). **(D)** Dot plot of biological processes related to the differentially expressed genes between the LPS and LPS + PtAu_2_ groups. **(E)** Chord plot of GO analysis of the differentially expressed genes between the LPS and LPS + PtAu_2_ groups. **(F)** KEGG pathway enrichment analysis of the differentially expressed genes between the LPS and LPS + PtAu_2_ groups. **(G)** GSEA of the differentially expressed genes between the LPS and LPS + PtAu_2_ groups.

To further elucidate the potential mechanisms underlying the anti-inflammatory and anti-OC properties of PtAu_2_ clusters, we performed RNA sequencing. Principal components analysis (PCA) revealed that the Control, LPS, and LPS + PtAu_2_ groups had different transcriptomic profiles (Figure 6**A**). The Venn diagram illustrates that the three groups shared 9341 genes, with 495, 56, and 85 genes exclusively expressed in the Control, LPS, and LPS + PtAu_2_ groups, respectively (Figure 6**B**). The Volcano plots displayed that 2672 genes were significantly upregulated with 2551 genes downregulated after LPS stimulation, while 128 genes were significantly upregulated and 109 genes were downregulated after treatment with PtAu_2_ clusters (fold change ≥ 2.0, *p* < 0.05) (Figure S22). As shown in Figure 6**C**, the clustered heat map shows that the expression of various pro-inflammatory genes, such as *Tnf*, *Nlrp3*, *Ptgs2*, *Mmp13*, *Il1b*, *Il12b*, *Il6*, and *Il23a*, was markedly elevated following LPS stimulation, whereas it was significantly reduced after treatment with PtAu_2_ clusters. Meanwhile, it was noted that the expression of a series of anti-inflammatory and anti-oxidative genes, including *Gstp1*, *Cat*, *Hmox1*, *Mgst2*, *Gclm*, and *Prdx1*, was upregulated after PtAu_2_ clusters treatment compared to the LPS group. GO enrichment analysis of the differentially expressed genes between the LPS and LPS + PtAu_2_ groups demonstrated significant changes in immune response, positive regulation of NF-κB transcription factor activity, and positive regulation of OC differentiation (Figure 6**D**). The GO analysis chord plot also revealed that treatment with PtAu_2_ clusters downregulated the expression of genes related to the positive regulation of OC differentiation (*Tnfα*, *Il12b*, *Il23a*), positive regulation of NO biosynthetic processes (*Klf2*, *Tnfα*, *Il1β*), and other inflammatory biological processes, but upregulated the expression of genes related to angiogenesis (*Hmox1*, *Angptl2*, *Plxdc1*), glutathione biosynthetic processes (*Gclm* and *Mgst2*), and other anti-inflammatory biological processes (Figure 6**E**). We also performed KEGG analysis of the differentially expressed genes between the LPS and LPS + PtAu_2_ groups to identify the top 20 enriched pathways, which included cytokine-cytokine receptor interactions, the TNF, NF-κB, and JAK-STAT signaling pathways (Figure 6**F**). In addition, gene set enrichment analysis (GSEA) revealed that genes related to cytokine-cytokine receptor interactions and OC differentiation were significantly enriched and downregulated after treatment with PtAu_2_ clusters, compared with the LPS group (Figure 6**G**). Taken together, these results suggest that PtAu_2_ clusters may exert anti-inflammatory effects by disrupting cytokine-cytokine receptor interactions, thereby inhibiting downstream signaling pathways such as the NF-κB and JAK-STAT signaling pathways. These findings are consistent with previous reports stating that gold clusters can inhibit the activation of cytokine receptors by cysteine residue-containing cytokine peptides through direct interactions between Au^+^ and thiol groups in the stimulating peptides 20, 45.

### PtAu_2_ clusters promote Nrf2 release from the Keap1/Nrf2 complex

**Figure 7:**
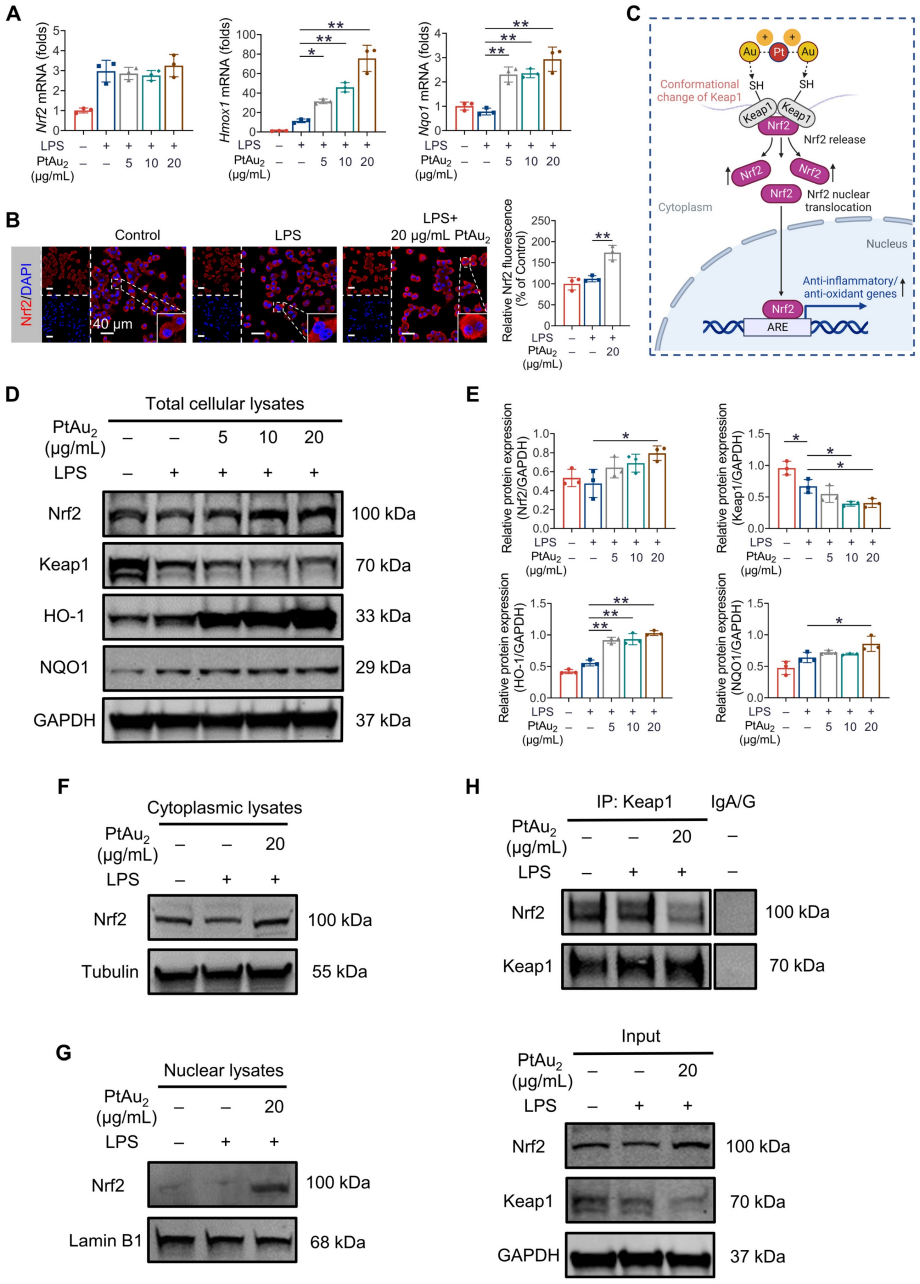
** PtAu_2_ clusters promote the release of Nrf2 from the Keap1/Nrf2 complex. (A)** RT-qPCR analysis of Nrf2 and downstream gene expression in LPS-activated RAW 264.7 macrophages treated with various concentrations of PtAu_2_ clusters. **(B)** Immunofluorescence analysis of Nrf2 in LPS-activated RAW 264.7 macrophages treated with various concentrations of PtAu_2_ clusters.** (C)** Schematic diagram of the modulation of Nrf2 and Keap1 conformation by PtAu_2_ clusters (created with BioRender.com). **(D)** Expression of Keap1/Nrf2 signaling members in total cellular lysates after treatment with various concentrations of PtAu_2_ clusters. **(E)** Quantitative analysis of western blotting results. **(F)** Nrf2 expression in cytoplasmic lysates. **(G)** Nrf2 expression in nuclear lysates. **(H)** Co-immunoprecipitation assays of Keap1-Nrf2. Data represent the mean ± SD of three independent experiments (one-way ANOVA with Tukey's post hoc test). *p < 0.05; **p < 0.01.

Since RNA sequencing analysis suggested that PtAu_2_ cluster treatment upregulated the expression of Nrf2 downstream genes with anti-inflammatory and anti-oxidative properties, including *Hmox1*, *Gclm*, *Cat*, *Prdx1*, *Mgst2*, and *Gstp1* (Figure 6**C**), we performed RT-qPCR analysis to verify these findings. As shown in Figure 7**A**, PtAu_2_ clusters markedly increased the mRNA expression of *Hmox1* and *Nqo1* in a dose-dependent manner without affecting that of *Nrf2*. Thus, PtAu_2_ clusters may upregulate the transcriptional expression of antioxidants downstream of Nrf2, to increase endogenous antioxidant production and reduce intracellular ROS. Nrf2 has been reported to bind to Keap1, which is anchored to the actin cytoskeleton and promotes Nrf2 degradation in unstimulated cells 48. The conformational changes in Keap1 induced by oxidative stress or chemicals with electrophilic properties allow Nrf2 to dissociate from Keap1 and translocate into the nucleus, where it increases the production of downstream antioxidants 49. To further explore the effect of PtAu_2_ clusters on Nrf2 activation, immunofluorescence was used to detect Nrf2 expression in LPS-challenged macrophages treated with PtAu_2_ clusters. As shown in Figure 7**B**, increased Nrf2 expression and nuclear translocation were observed in LPS-challenged macrophages treated with PtAu_2_ clusters compared to LPS-challenged macrophages. Considering the strong interaction reported between Au^+^ and thiol groups, we speculated that PtAu_2_ clusters might activate Nrf2 expression by modifying cysteine residues in Keap1, leading to a conformational change that promotes the release and nuclear translocation of Nrf2 and the upregulation of downstream anti-inflammatory and anti-oxidative genes (Figure 7**C**).

To verify this hypothesis, we evaluated the protein expression of Keap1, Nrf2, and its downstream antioxidants by western blotting of total cellular lysates of LPS-challenged macrophages treated with PtAu_2_ clusters. As expected, PtAu_2_ clusters downregulated Keap1 expression but upregulated the expression of Nrf2 and its downstream antioxidants, including HO-1 and NQO1, in a dose-dependent manner (Figures 7**D** and** 7E**). Similar results of increased expression of Nrf2 and its downstream HO-1 and NQO1 could also be observed in RANKL-stimulated BMMs with PtAu_2_ clusters treatment (Figure S23). Furthermore, cytoplasmic Nrf2 expression was upregulated by PtAu_2_ clusters, indicating increased Nrf2 protein stabilization and accumulation (Figure 7**F**). Consistent with the immunofluorescence results, PtAu_2_ clusters increased nuclear translocation of Nrf2, suggesting increased Nrf2 activation (Figures 7**G** and S24). To elucidate the underlying mechanism, we examined whether PtAu_2_ clusters affect the association between Keap1 and Nrf2 in LPS-activated macrophages. Co-immunoprecipitation assays confirmed the low levels of immunoprecipitation between Keap1 and Nrf2 following treatment with PtAu_2_ clusters (Figure 7**H**). In addition, we also explored whether PtAu_2_ clusters affect other endogenous anti-inflammatory and anti-oxidative pathways by RT-qPCR (Figure S25). The results demonstrated that PtAu_2_ clusters hardly affected gene expression of *Foxo1*, *Sirt1*, and *Ppargc1a* in LPS-stimulated macrophages, indicating that these signaling molecules may not be involved in the anti-inflammatory and anti-oxidative efficacy of PtAu_2_ clusters. Taken together, these findings suggest that PtAu_2_ clusters disrupt the association between Keap1 and Nrf2, thereby promoting the release and nuclear translocation of Nrf2 as well as the activation of endogenous anti-inflammatory and anti-oxidative products.

### Inhibitory effect of PtAu_2_ clusters on LPS-induced calvarial osteolysis *in vivo*

**Figure 8:**
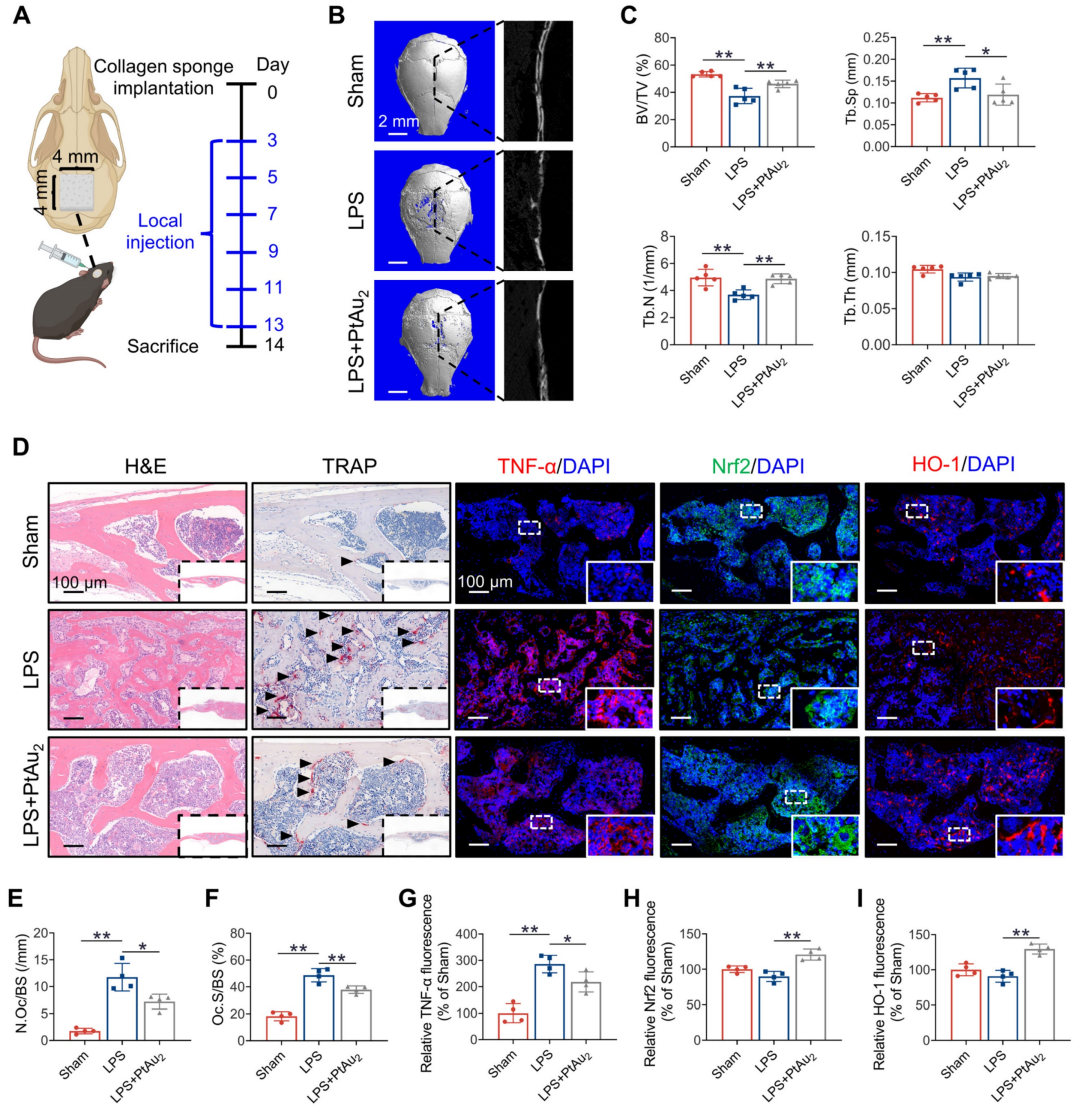
** Inhibitory effect of PtAu_2_ clusters on LPS-induced calvarial osteolysis *in vivo*. (A)** Schematic diagram of *in vivo* experiments. **(B)** Representative micro-CT scanning images of mice calvaria. **(C)** Quantitative analysis of micro-CT scanning images. **(D)** Representative H&E, TRAP, and immunostaining images of TNF-α, Nrf2, and HO-1. **(E)** Quantification of the number of TRAP-positive OCs. **(F)** Quantification of OcS/BS%.** (G)** Quantitative analysis of relative TNF-α fluorescence. **(H)** Quantitative analysis of relative Nrf2 fluorescence. **(I)** Quantitative analysis of relative HO-1 fluorescence. **(C)** Data represent the mean ± SD of five independent animals (one-way ANOVA with Tukey's post hoc test). **(E, F, G, H, and I)** Data represent the mean ± SD of four independent animals (one-way ANOVA with Tukey's post hoc test). *p < 0.05; **p < 0.01.

Having determined the biocompatibility, cellular uptake, anti-inflammatory phenotypes, and anti-inflammatory mechanisms of PtAu_2_ clusters *in vitro*, we investigated their therapeutic efficiency *in vivo* by establishing a murine model of LPS-induced calvarial osteolysis. As shown in Figure 8**A**, LPS-soaked collagen sponge blocks were implanted into mouse calvaria to cause inflammation-induced bone loss, and local PBS or PtAu_2_ injections were administered every other day. After 14 days, the mouse calvaria were harvested for radiographic and histological assessment. Micro-CT revealed extensive bone erosion on the calvarial bone surface in the LPS group, with numerous large and deep resorption pits (Figure 8**B**). In contrast, PtAu_2_ clusters significantly reduced the scope and extent of bone destruction, as evidenced by a reduction in the number of resorption pits. Quantitative analysis also illustrated that key morphometric parameters, including bone volume/tissue volume (BV/TV), trabecular bone number (Tb.N), and trabecular bone separation (Tb.Sp), were markedly improved in the PtAu_2_ group compared to the LPS group (Figure 8**C**).

Histological evaluation confirmed the protective effect of PtAu_2_ clusters on bone resorption and inflammatory reactions *in vivo*. Consistent with the micro-CT findings, H&E staining revealed that the local LPS injection induced extensive osteolysis, whereas PtAu_2_ clusters alleviated this effect (Figure 8**D**). In addition, TRAP staining demonstrated that the number of TRAP-positive OCs was increased by LPS injection but reduced by PtAu_2_ clusters (Figures 8**E** and 8**F**). Immunofluorescence staining further suggested that LPS significantly increased TNF-α expression, whereas PtAu_2_ clusters markedly reduced TNF-α expression (Figure 8**G**). Consistent with the *in vitro* results, both *in vivo* western blotting and immunofluorescence staining illustrated that PtAu_2_ clusters effectively activated Nrf2 expression (Figure S26 and 8**H**). Consistent with these results, immunofluorescence staining revealed that PtAu_2_ clusters elevated HO-1 expression, an important downstream anti-oxidative product of Nrf2 (Figure 8**I**). Collectively, these results indicate that PtAu_2_ clusters can attenuate LPS-induced osteolysis by inhibiting bone resorption and inflammatory activity, and activating endogenous anti-inflammatory systems.

Moreover, we examined the biosafety of PtAu_2_ clusters. H&E staining revealed no obvious unfavorable effects on the histological features of the major organs, including the heart, liver, spleen, lungs, and kidneys (Figure S27). Meanwhile, complete blood panel analysis and serum biochemistry analysis also demonstrated that PtAu_2_ clusters did not exhibit toxicity 11 days after PtAu_2_ clusters treatment (Figure S28). Furthermore, ICP-MS was performed to evaluate the biodistribution and metabolism of PtAu_2_ clusters 48 h after administration. The results showed that the liver, spleen, and intestine exhibited the highest dosage distribution (Figure S29), which is consistent with a previous report on the biodistribution of bimetallic clusters 30, indicating possible hepatobiliary or fecal elimination of PtAu_2_ clusters. Together, these results suggest a satisfactory level of biosafety as well as an excretion pathway of PtAu_2_ clusters.

## Conclusions

In this study, we demonstrated that PtAu_2_ nanoclusters with electron-rich 4-ethynyl benzene-1,2-diamine show specifical NO-responsive phosphorescent properties and exhibit strong binding interactions with cysteine, thereby qualifying them as sensitive inflammation detectors, as well as an ideal anti-inflammatory agent. The potent anti-inflammatory effects of PtAu_2_ clusters can be attributed to the following mechanisms: i) interrupting cytokine-cytokine receptor interactions to inhibit downstream inflammatory signaling pathways; and ii) activating Nrf2 expression by disrupting the Keap1-Nrf2 association, causing increased Nrf2 release and nuclear translocation to upregulate the expression of endogenous anti-inflammatory and anti-oxidative products. Furthermore, *in vivo* experiments confirmed that PtAu_2_ clusters alleviated inflammatory osteolysis by inhibiting osteoclastogenesis and increasing Nrf2 expression, despite the molecular structural basis of PtAu_2_ clusters binding to their targeted proteins remains to be investigated by experimental crystal structure studies. From the perspective of clinical translation, PtAu_2_ clusters could be directly used as therapeutic agents or coatings for implants, or in combination therapies with other drugs to reduce systemic toxicity, while these nanoclusters may possess certain limitations, including inadequate ROS-scavenging properties and a lack of responsiveness to various stimuli. Nevertheless, by developing novel heterometallic nanoclusters as activators of endogenous anti-inflammatory systems, this study provides new insights into the use of multifunctional molecular therapeutic agents for the treatment of inflammatory osteolysis and other inflammatory diseases.

## Supplementary Material

Supplementary materials and methods, figures and tables.

## Abbreviations

ANOVA: one-way analysis of variance
CCK-8: cell Counting Kit-8
CLSM: confocal laser scanning microscopy
DHE: dihydroethidium
DMSO: dimethyl sulfoxide
FBS: fetal bovine serum
GSH/GSSG: glutathione/glutathione disulfide
GO: Gene ontology
GSEA: gene set enrichment analysis
HRMS: high-resolution mass spectrometry
IL: interleukin
iNOS: inducible nitric oxide synthase
Keap1: Kelch like ECH associated protein 1
KEGG: Kyoto encyclopedia of genes and genomes
LPS: lipopolysaccharide
M-CSF: macrophage colony stimulating factor
α-MEM: minimal essential medium alpha
NSAIDs: non-steroidal anti-inflammatory drugs
NO: nitric oxide
Nrf2: nuclear factor erythroid 2-related factor 2
OCs: osteoclasts
PBS: phosphate buffered saline
PFA: paraformaldehyde
PVDF: polyvinylidene fluoride
PCA: principal components analysis
ROS: reactive oxygen species
RANKL: receptor activator of NF-κB ligand
SMT: methylisothiourea sulfate
TNF-α: tumor necrosis factor-α
TJR: total joint replacement
TRAP: tartrate resistant acid phosphatase
