# Supplementary material for: Ultrasmall PtAu2 nanoclusters activate endogenous anti-inflammatory and anti-oxidative systems to prevent inflammatory osteolysis
Source: Theranostics. 2023 Jan 22;13(3):1010–27. doi: 10.7150/thno.80514 (PMC9925309; doi:10.7150/thno.80514)
Supplement: Supplementary file 1 — Supplementary materials and methods, figures and tables. [file thnov13p1010s1.pdf]

Supporting Information

Ultrasmall $PtAu_2$ nanoclusters activate endogenous anti-inflammatory and anti-oxidative systems to prevent inflammatory osteolysis

Xuzhuo Chen[a,1], Xiankun Cao[c,1], Dasheng Zheng[b], Chang Li[b], Yan Chen[c], Keyu Kong[c], Weifeng Xu[a], Bin Shi[d], Xinwei Chen[a,*], Fengrong Dai[b,*], Shanyong Zhang[a,*]

[a]*Department of Oral Surgery, Shanghai Key Laboratory of Stomatology & Shanghai Research Institute of Stomatology, National Clinical Research Center for Oral Diseases, Shanghai Ninth People's Hospital, College of Stomatology, Shanghai Jiao Tong University School of Medicine, Shanghai, 200011, China*

[b]*State Key Laboratory of Structural Chemistry, Fujian Institute of Research on the Structure of Matter, Chinese Academy of Sciences, Fuzhou, Fujian, 350002, China*

[c]*Department of Orthopedics, Shanghai Key Laboratory of Orthopedic Implant, Shanghai Ninth People's Hospital, Shanghai Jiao Tong University School of Medicine, Shanghai, 200011, China*

[d]*Department of Oral and Maxillofacial Surgery, The First Affiliated Hospital, Fujian Medical University, Fuzhou 350002, Fujian, China*

[*]Corresponding authors.

E-mail addresses: chenxinwei@sjtu.edu.cn (XW. Chen), dfr@fjirsm.ac.cn (FR. Dai), zhangshanyong@126.com (SY. Zhang).

[1]These authors contributed equally.

The supporting information includes:

- Supplementary Methods
- Supplementary Figures
- Supplementary Tables

Supplementary Methods

Synthesis of A

Ethynyltrimethylsilane (1.96 g, 20 mmol), $Pd(PPh_3)_4$ (230 mg, 0.2 mmol), $Pd(PPh_3)_2Cl_2$ (280 mg, 0.4 mmol), and CuI (76 mg, 0.4 mmol) were added to triethylamine (40 mL) solution of 1,2-diamine-4-iodobenzene (2.34 g, 10 mmol) with stirring at room temperature for 2 h. Upon heating at 75 ^oC for 12 h, the solution was taken by filtration and the filtrate was collected. The product was purified by a silica gel column chromatography using petroleum-CH_2Cl_2 (v/v = 5:1) as an eluent to give a colorless solid. Yield: 86%. ^1H NMR (400 MHz, $CDCl_3$, ppm): δ 6.88 (d, *J* = 7.8 Hz, 1H), 6.84 (s, 1H), 6.59 (d, J = 7.8 Hz, 1H), 3.26 (s, 4H), 0.22 (s, 9H).

Synthesis of B

Under nitrogen atmosphere, **A** (1.63 g, 8.0 mmol) was first dissolved in 50 mL ethanol. To the solution were added di-tert-butyl dicarbonate (3.92 g, 18.0 mmol) and Na_2CO_3 (1.90 g, 18.0 mmol). After stirring at room temperature for 4 h, it was filtered and the concentrated filtrate was

put into a silica gel column. The product was purified by column chromatography using petroleum-CH_2Cl_2 (v/v = 5:1) as an eluent to give a colorless solid. Yield: 72%. ^{1}H NMR (400 MHz, $CDCl_3$, ppm): δ 7.58–7.52 (m, 2H), 7.23 (d, J = 8.4 Hz, 1H), 6.80 (s, 1H), 6.60 (s, 1H), 1.51 (s, 18H), 0.22 (s, 9H).

Synthesis of C

To a $CHCl_3$ (200 mL) solution of $Pt(PPh_3)_2Cl_2$ (395 mg, 0.5 mmol) were added **B** (0.444 g, 1.1 mmol), Et_3N (0.15 mL, 1.1 mmol) and CuI (2 mg). The reaction solution was heated at 65 ^oC for 12 h, and then the solvents were removed under a vacuum. The product was purified by silica gel column chromatography using CH_2Cl_2-CH_3OH (v/v = 50:1) as an eluent to give a yellow solid. Yield: 68%. ^{1}H NMR (400 MHz, $CDCl_3$, ppm): δ 7.78 (dd, J = 11.8, 5.8 Hz, 12H), 7.45 (dd, J = 11.2, 7.8 Hz, 2H), 7.41 – 7.32 (m, 18H), 7.14 (d, J = 7.5 Hz, 2H), 6.72 (s, 1H), 6.67 (s, 1H), 6.16 (s, 1H), 6.05 (d, J = 7.8 Hz, 1H), 1.47 (d, J = 12.1 Hz, 36H). ^{31}P NMR (162 MHz, $CDCl_3$, ppm): 18.6 (s, 2P, $J_{Pt\text{-}P}$ = 2648 Hz).

Synthesis of BOC-protective PtAu$_2$-1a cluster

Au(tht)Cl (128 mg, 0.4 mmol), dTolmp (224 mg, 0.4 mmol), KSO_3CF_3 (76 mg, 0.4 mmol), and dry CH_2Cl_2 (20 mL) were added to a 50 mL Schlenk flask with stirring for 0.5 h. To this Schlenk flask was slowly added a CH_2Cl_2 (10 mL) solution of **C** (276 mg, 0.20 mmol). Upon stirring at room temperature for 4 h, the solution was evaporated to dryness under reduced pressure. The product was purified by silica gel column chromatography using CH_2Cl_2-MeOH (v/v = 25:1) as eluent to afford the product as a yellow solid. Yield: 62%. ^{1}H NMR (400 MHz, CD_3OD, ppm) δ 7.99 (dd, J = 13.4, 6.6 Hz, 5H), 7.78 (dd, J = 13.3, 6.5 Hz, 5H), 7.52 (t, J = 7.5 Hz, 5H), 7.39 (t,

J = 7.3 Hz, 7H), 7.33 (d, J = 7.7 Hz, 7H), 7.16 (t, J = 7.5 Hz, 4H), 7.10 (t, J = 7.5 Hz, 3H), 6.97 (d, J = 7.4 Hz, 4H), 6.91 (t, J = 7.7 Hz, 4H), 6.78 (d, J = 8.3 Hz, 2H), 6.74 (t, J = 7.6 Hz, 4H), 4.78 – 4.59 (m, 8H), 2.31 (s, 12H), 2.14 (s, 12H), 1.58 (d, J = 8.5 Hz, 36H). ^{31}P NMR (162 MHz, CD_3OD, ppm): 18.43 (t, 4P, $J_{P–P}$ = 32 Hz), 5.6-2.7 (m, 2P, $J_{Pt\text{-}P}$ = 2536 Hz, $J_{P\text{-}P}$ = 32 Hz). HRMS (ESI): m/z calculated for $[M—2SO_3CF_3]^{2+}$: 1188.3248; Found: 1188.3246. Crystallographic data and structure refinement are summarized in Table S2.

Synthesis of PtAu$_2$-1 cluster

To a MeOH (20 mL) of PtAu$_2$-**1a** cluster (238 mg, 0.1 mmol) was added $(CH_3)_3SiI$ (90 mg, 0.45 mmol). Upon stirring at room temperature for 2 h, the product was purified by silica gel column chromatography using CH_2Cl_2-MeOH (v/v = 10:1) as eluent to afford the product as a brown solid. Yield: 45%. ^{1}H NMR (400 MHz, CD_3OD, ppm) δ 8.33 (s, 2H), 8.09 (s, 2H), 7.97 (s, 2H), 7.91 – 7.77 (m, 4H), 7.52 (d, J = 5.2 Hz, 4H), 7.46 – 7.29 (m, 11H), 7.25 – 7.03 (m, 11H), 6.94 (d, J = 12.5 Hz, 7H), 6.68 (t, J = 7.5 Hz, 2H), 6.54 (d, J = 8.7 Hz, 2H), 6.39 (d, J = 7.1 Hz, 1H), 5.19 – 4.54 (m, 8H), 2.32 (s, 6H), 2.22 (s, 6H), 2.15 (s, 12H). ^{31}P NMR (400 MHz, CD_3OD, ppm) δ 21.2 (s, 4P, $J_{P–P}$ = 32 Hz), 5.52 (m, 2P, $J_{Pt\text{-}P}$ = 2522 Hz, $J_{P\text{-}P}$ = 32 Hz). HRMS (ESI): m/z calculated for $[M—2SO_3CF_3]^{2+}$: 988.2144; Found: 988.2183.

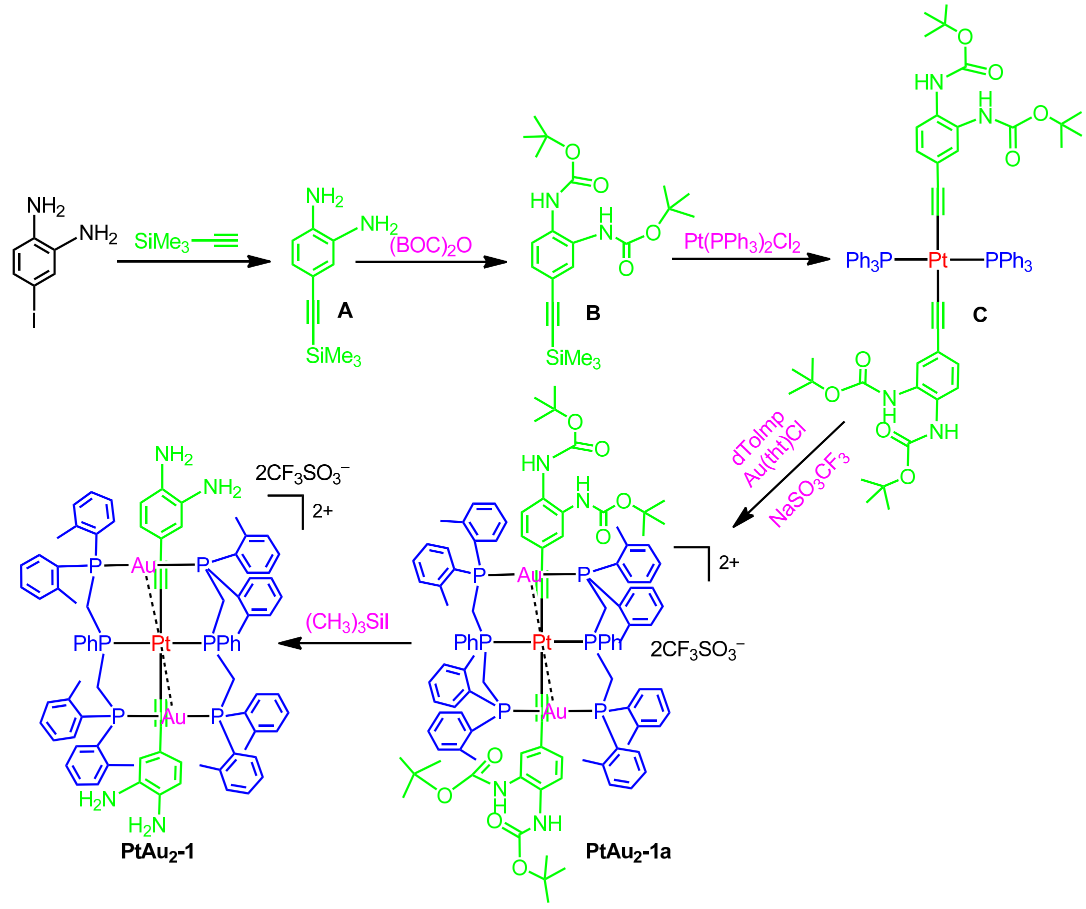

Scheme S1. Synthetic procedures of PtAu$_2$-**1** cluster.

Cell culture

RAW 264.7 murine macrophages were kindly provided by Cell Bank/Stem Cell Bank, Chinese Academy of Sciences. The cells were cultured in complete α-MEM supplemented with 10% FBS, 100 U/mL penicillin, and streptomycin at 37 °C with 5% CO_2. The cells were ready to be seeded or passaged when the confluence reached 80–90%. To avoid additional stimuli to the macrophage cell line, scrapers were used to remove the attached cells instead of trypsin, in the process of cell dissociation. The murine primary bone marrow macrophages (BMMs) were obtained and cultured as the previous protocols [1]. Briefly, the cells were isolated from the femurs

and tibiae of 4-week-old C57/BL6 male mice, cultured in complete α-MEM with 30 ng/mL M-CSF at 37 °C with 5% CO_2. When the confluence reached 80–90%, the cells were ready to be seeded and dissociated by 0.25% trypsin-EDTA (Gibco, Thermo Fisher Scientific, Waltham, MA, USA).

Cytotoxicity assay

For live/dead staining, RAW 264.7 murine macrophages were treated with various concentrations of $PtAu_2$ clusters for 24 h. After that, the cells were incubated with the PI solution and Calcein-AM for 15 min. Then the live/dead cells were observed via confocal laser scanning microscopy (CLSM, Leica TCS-SP5, DM6000-CFS). The results were quantified by calculating the percent positive cells of Calcein-AM and PI, respectively.

For the CCK-8 assay, RAW 264.7 murine macrophages and BMMs were seeded into 96-well plates at a density of 8×10^3 cells/well, respectively. The cells were treated with various concentrations of $PtAu_2$ clusters for the corresponding time (24 and 48 h for RAW 264.7 macrophage; 48 and 96 h for BMMs). 10 μL of CCK-8 solution was added to each well at every time point. After 2 h incubation, the absorbance was measured at the wavelength of 450 nm, with 630 nm as the reference wavelength in a microplate reader. The results were presented as cell viability relative to the control group, which was set at 100%.

Cell cycle analysis

For evaluation of cell cycle distribution, RAW 264.7 murine macrophages were incubated with various concentrations of $PtAu_2$ clusters for 24 h. After treatment, cells were collected and fixed in cold 70% ethanol at 4 °C for 30 min. Then the cells were centrifuged at 1000 rpm for 5

min and incubated with 50 μg/mL PI (Beyotime, Shanghai, China) for 30 min. Subsequently, the cell cycle was detected by a FACScan flow cytometer (BD, CA, USA) for at least 20,000 cells per sample, then analyzed by FlowJo software.

Hemolysis assay

For the hemolysis assay, fresh blood was obtained from 4-week-old C57/BL6 male mice in anticoagulated tubes. The collected samples were centrifuged and washed until the supernatant was clear. After dilution with PBS, the red blood cell (RBC) suspension was added into EP tubes with various concentrations of $PtAu_2$ clusters in the same volume. Double distilled water and PBS were used as the positive and negative controls, respectively. After 2 h incubation at 37 °C, the tubes were centrifugated at 3,000 rpm for 5 min. The supernatant absorbance was measured at 540 nm by a microplate reader. The calculation of the hemolysis rate was shown as follows: Hemolysis rate (%) = $(OD_{sample} - OD_{negative})/(OD_{positive} - OD_{negative}) \times 100\%$.

Supplementary Figures

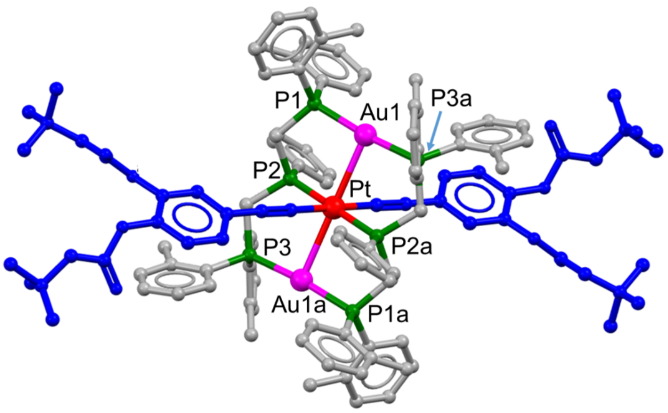

Figure S1. A perspective view of BOC-protective PtAu2-**1a** cluster, plotted from X-ray crystallography.

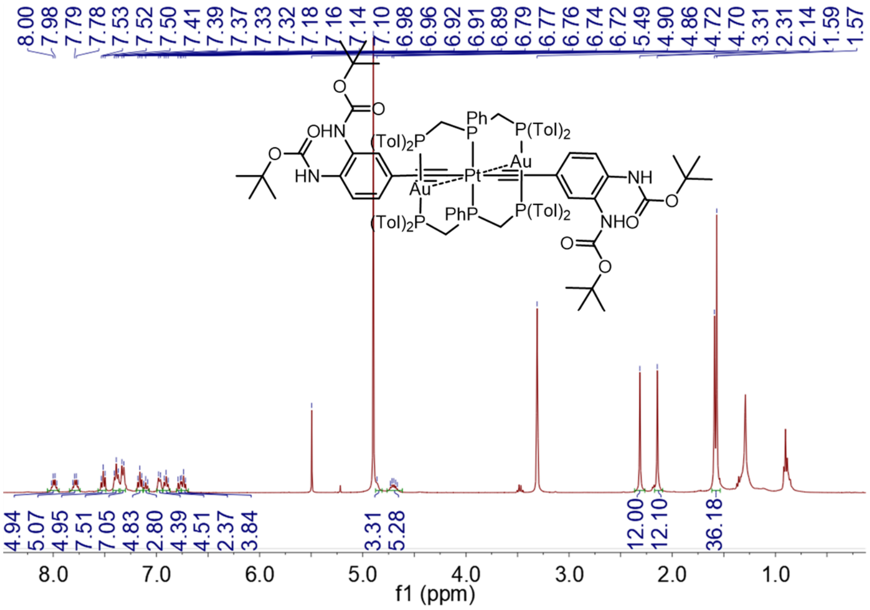

Figure S2. The ^{1}H NMR spectrum of BOC-protective PtAu2-**1a** cluster in CD3OD at room temperature.

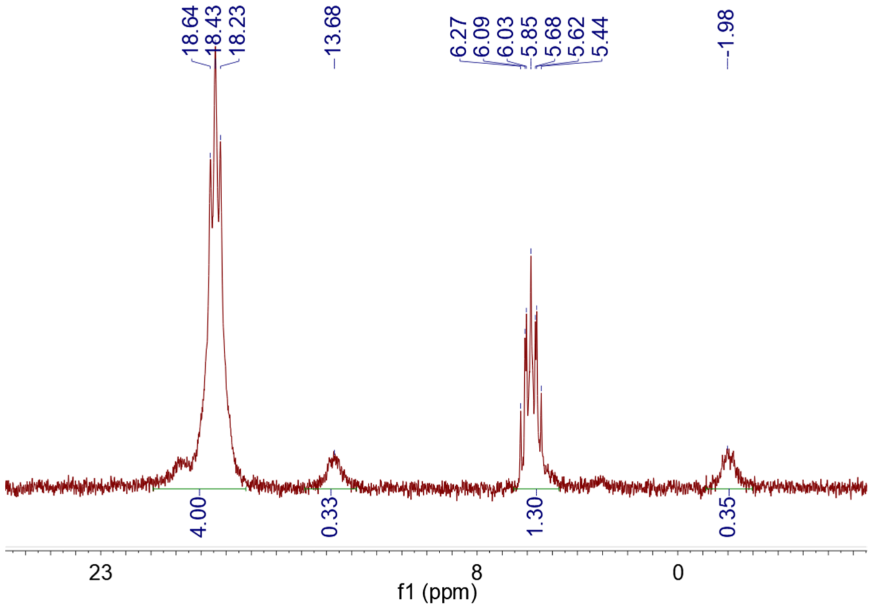

Figure S3. The ^{31}P NMR spectrum of BOC-protective $PtAu_2$-**1a** cluster in CD_3OD at room temperature.

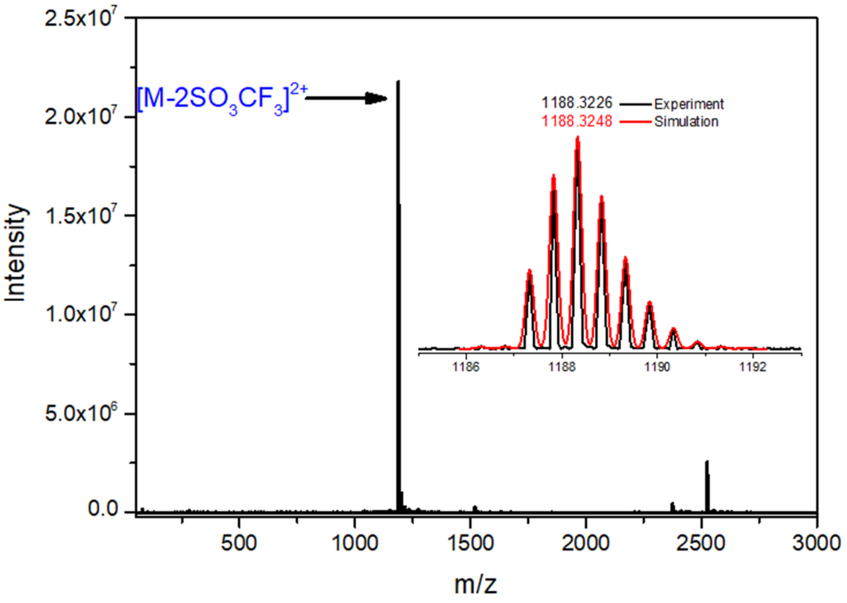

Figure S4. The high-resolution mass spectrometry (HRMS) of BOC-protective $PtAu_2$-**1a** cluster.

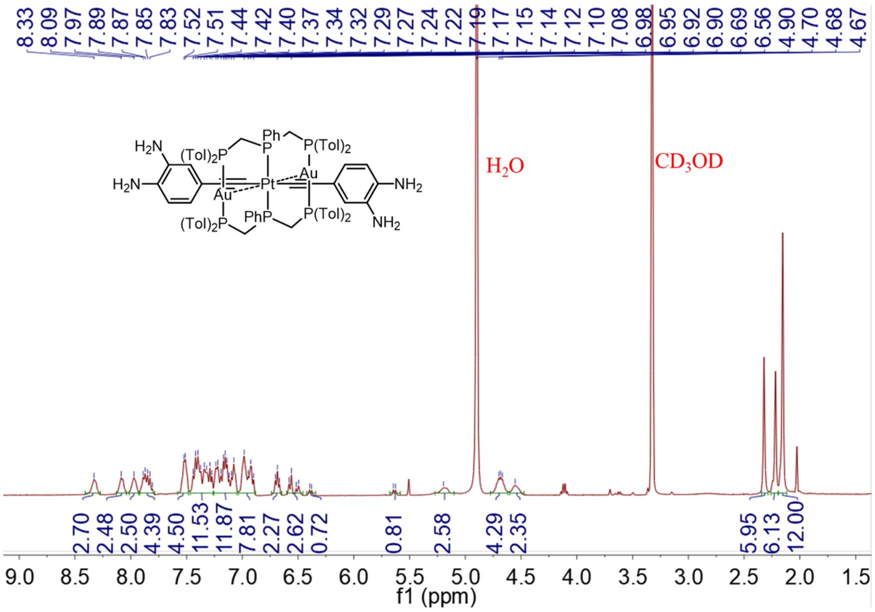

Figure S5. The ^{1}H NMR spectrum of $PtAu_2$-**1** cluster in CD_3OD at room temperature.

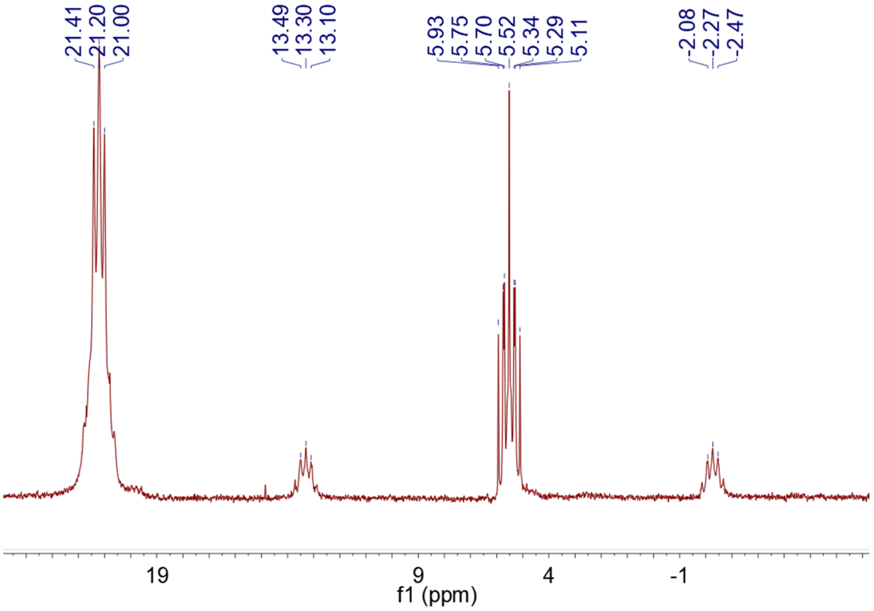

Figure S6. The ^{31}P NMR spectrum of $PtAu_2$-**1** cluster in CD_3OD at room temperature.

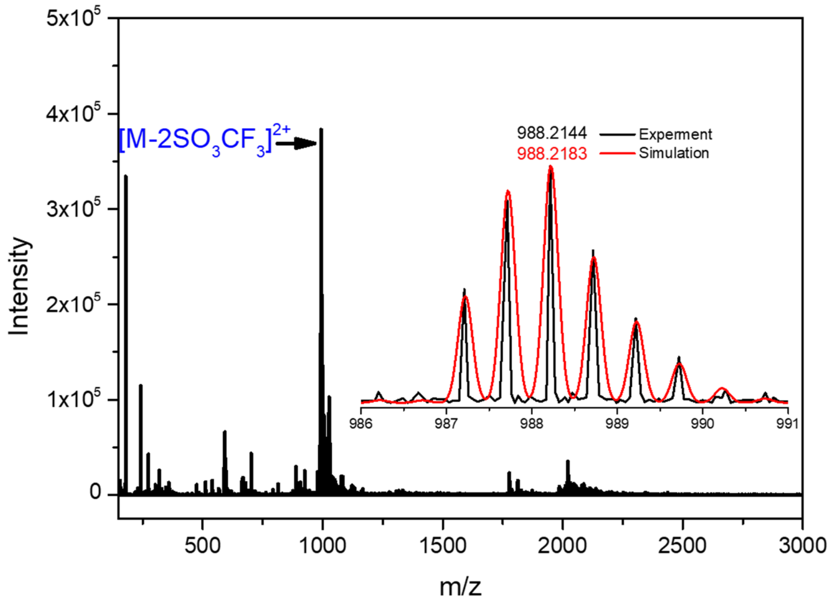

Figure S7. The high-resolution mass spectrometry (HRMS) of $PtAu_2$-**1** cluster.

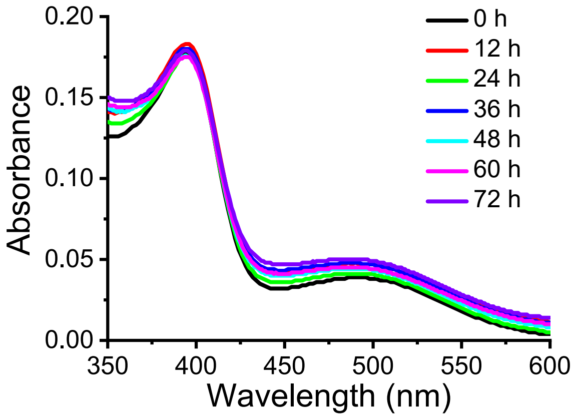

Figure S8. The UV-Vis absorption spectra of $PtAu_2$-**1** cluster in DMSO-PBS (v/v = 1:1) solution at a concentration of 1.0×10^{-5} M, measured at 0, 12, 24, 36, 48, 60 and 72 h, respectively.

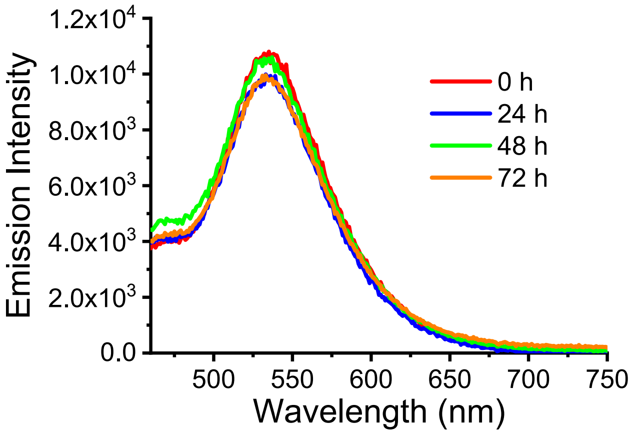

Figure S9. The phosphorescent spectra of $PtAu_2$-**1** cluster in DMSO-PBS (v/v = 1:1) solution at a concentration of 1.0×10^{-5} M upon excitation at 397 nm, measured at 0, 24, 48, and 72 h, respectively.

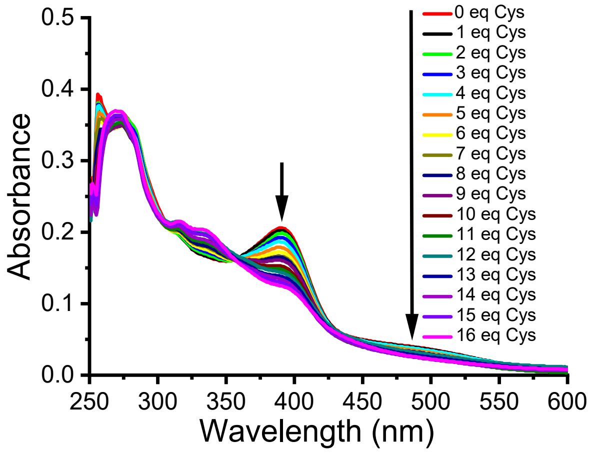

Figure S10. The UV-Vis absorption spectral change of $PtAu_2$-**1** cluster (1.0×10^{-5} M) in DMSO-PBS (v/v = 1 :1) solution upon the addition of an aqueous solution of L-cysteine, showing the gradual decrease of the peak at 390 nm.

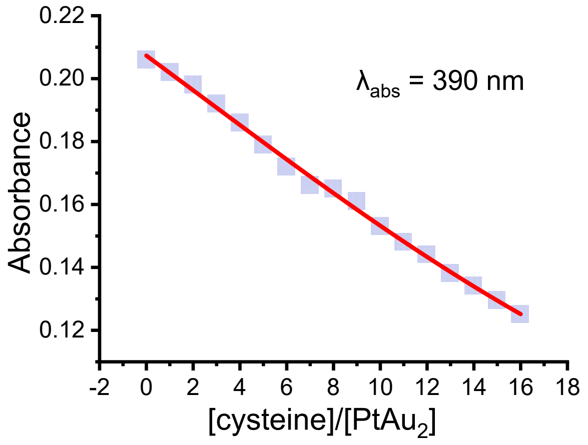

Figure S11. The dependence of absorbance at 390 nm on the molar ratio between L-cysteine *vs* $PtAu_2$-**1** cluster.

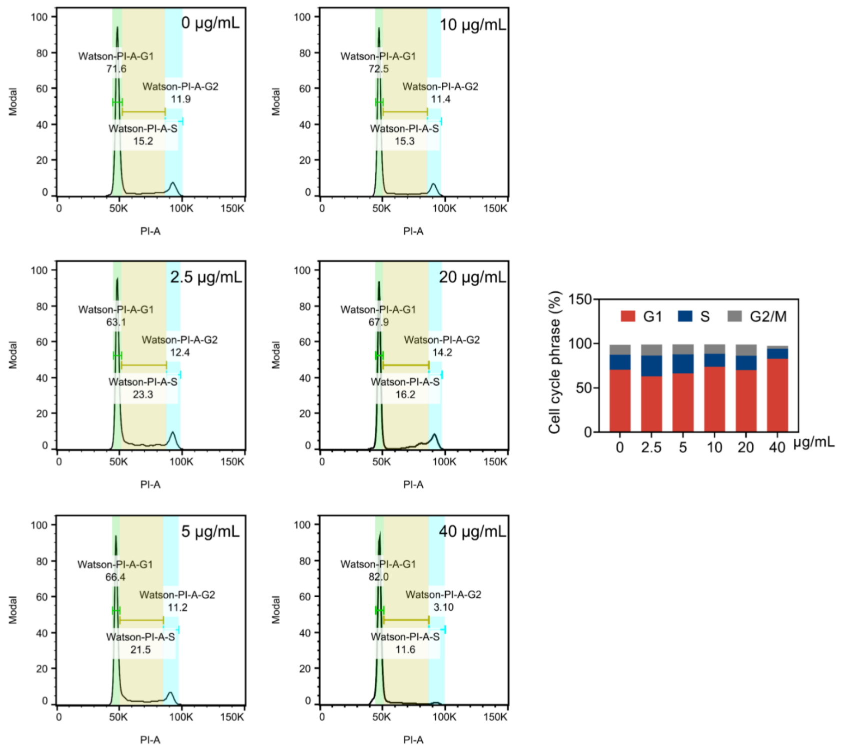

Figure S12. Cell cycle analysis of RAW 264.7 macrophages incubated with various concentrations of $PtAu_2$ clusters for 24 h.

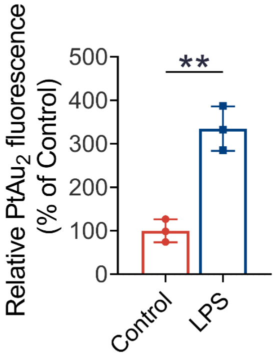

Figure S13. Percentage of $PtAu_2$ fluorescence intensity relative to the Control group. Data represent means ± SD from three independent replicates (unpaired two-tailed Student's t-test). ***p* < 0.01.

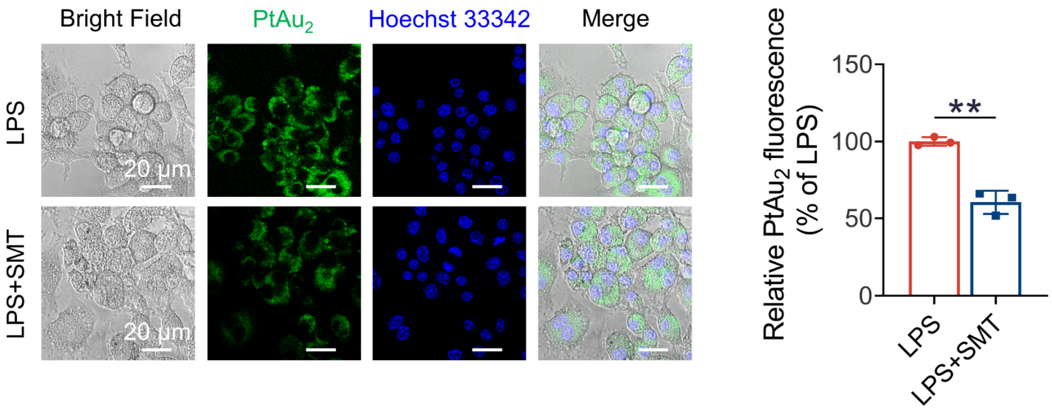

Figure S14. Confocal images of LPS-activated RAW 264.7 macrophages incubated with $PtAu_2$ clusters, with or without SMT. Data represent means ± SD from three independent replicates (unpaired two-tailed Student's t-test). ***p* < 0.01.

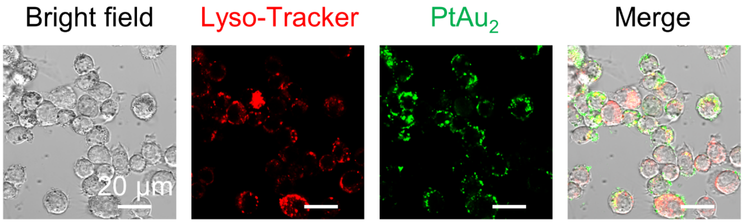

Figure S15. Confocal images of RAW 264.7 macrophages incubated with LPS plus $PtAu_2$ clusters for 12 h. Lyso-Tracker was used for staining of endo/lysosomes.

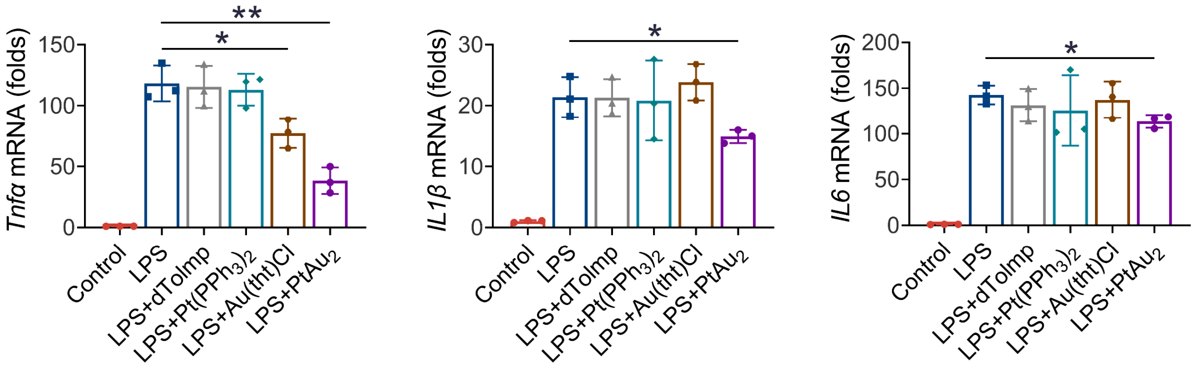

Figure S16. RT-qPCR analysis of pro-inflammatory gene expression in LPS-activated RAW 264.7 macrophages treated with various precursors of $PtAu_2$ clusters. Data represent the mean ± SD of three independent experiments (one-way ANOVA with Tukey's post hoc test). *$p < 0.05$; **$p < 0.01$.

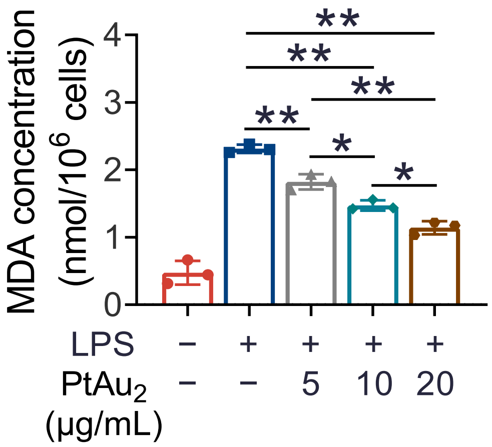

Figure S17. Intracellular MDA levels in LPS-activated RAW 264.7 macrophages treated with various concentrations of $PtAu_2$ clusters. Data represent the mean ± SD of three independent experiments (one-way ANOVA with Tukey's post hoc test). *$p < 0.05$; **$p < 0.01$.

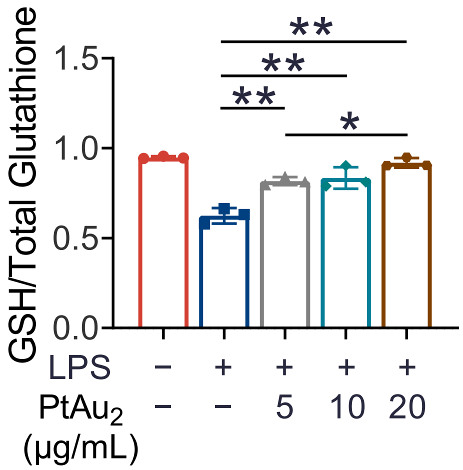

Figure S18. Intracellular GSH/total glutathione levels in LPS-activated RAW 264.7 macrophages treated with various concentrations of $PtAu_2$ clusters. Data represent the mean ± SD of three independent experiments (one-way ANOVA with Tukey's post hoc test). *$p < 0.05$; **$p < 0.01$.

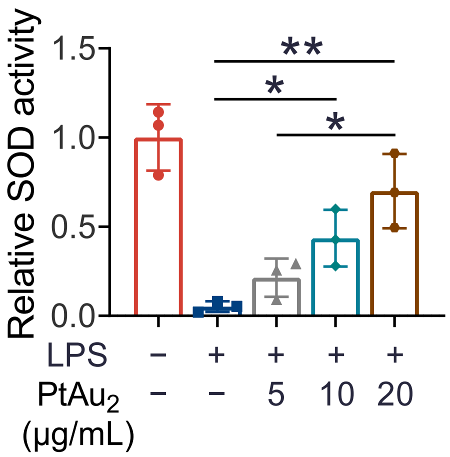

Figure S19. Relative SOD activity in LPS-activated RAW 264.7 macrophages treated with various concentrations of $PtAu_2$ clusters. Data represent the mean ± SD of three independent experiments (one-way ANOVA with Tukey's post hoc test). *$p < 0.05$; **$p < 0.01$.

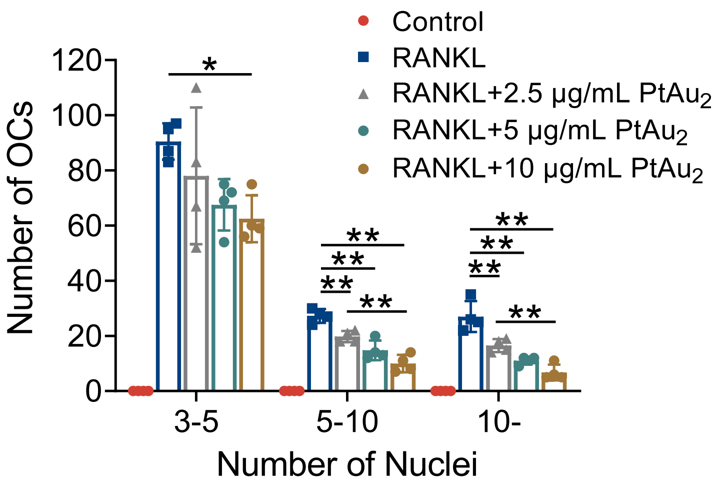

Figure S20. Quantification of number of OCs based on number of nuclei. Data represent the mean ± SD of four independent experiments (one-way ANOVA with Tukey's post hoc test).

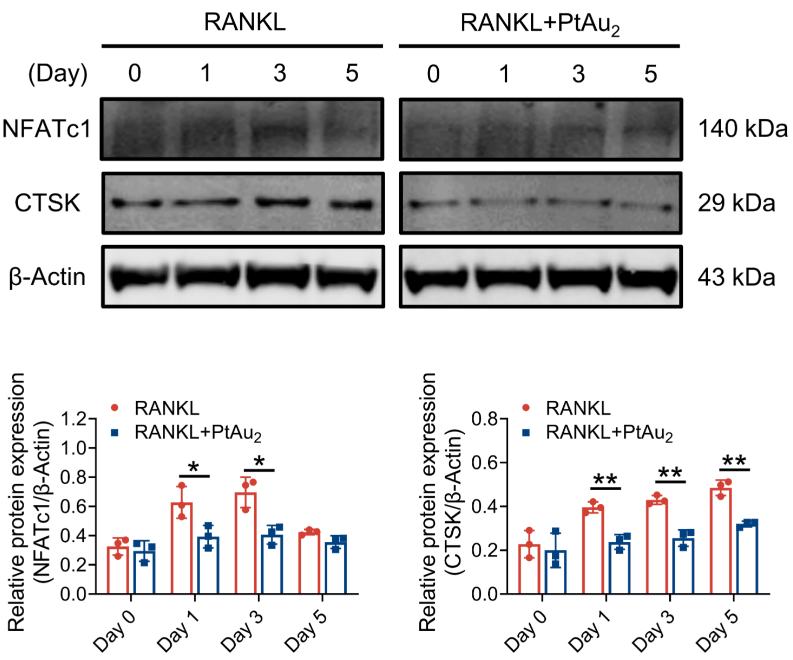

Figure S21. Expression of NFATc1 and CTSK in RANKL-stimulated BMMs treated with or without $PtAu_2$ clusters (10 μg/mL) for 0, 1, 3, and 5 days. Data represent the mean ± SD of three independent replicates (one-way ANOVA with Tukey's post hoc test). $^*p < 0.05$; $^{**}p < 0.01$.

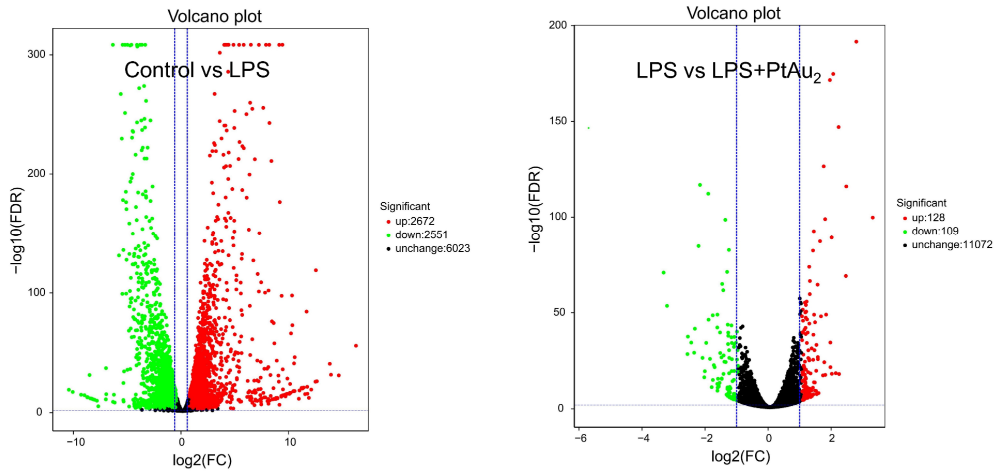

Figure S22. Volcano plots of the differentially expressed genes.

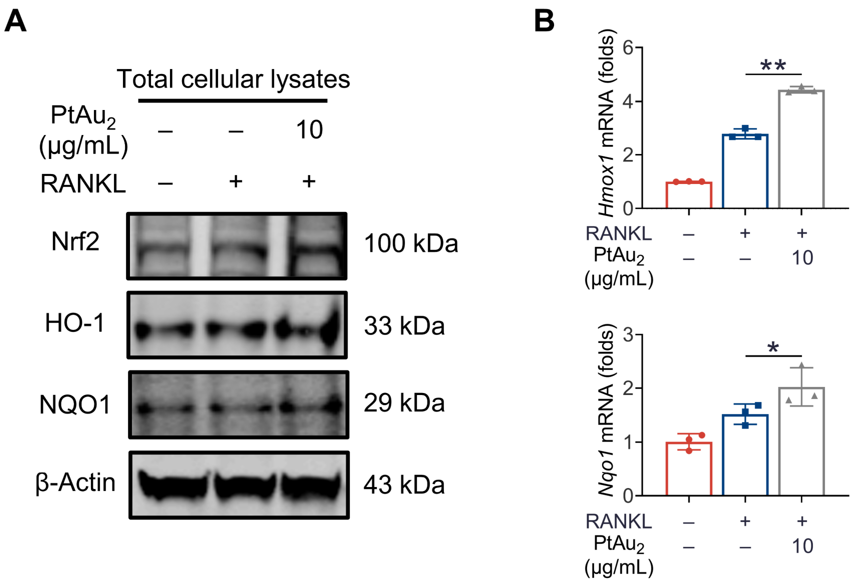

Figure S23. Expression of Keap1/Nrf2 signaling members in RANKL-stimulated BMMs treated with or without $PtAu_2$ clusters (10 μg/mL) for 4 days. (A) Western blotting analysis of Nrf2, HO-1, and NQO1. (B) RT-qPCR analysis of *Hmox1* and *Nqo1*. Data represent the mean ± SD of three independent replicates (one-way ANOVA with Tukey's post hoc test). $*p < 0.05$; $**p < 0.01$.

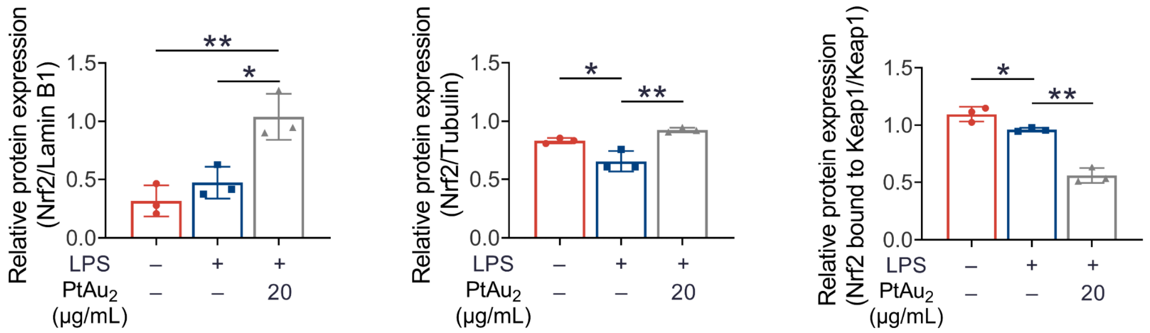

Figure S24. Quantitative analysis of nuclear and cytoplastic protein expression of Nrf2, and protein expression of Nrf2 bound to Keap1/Keap1. Data represent means ± SD from three independent replicates (one-way ANOVA with Tukey's post hoc tests). $*p < 0.05$; $**p < 0.01$.

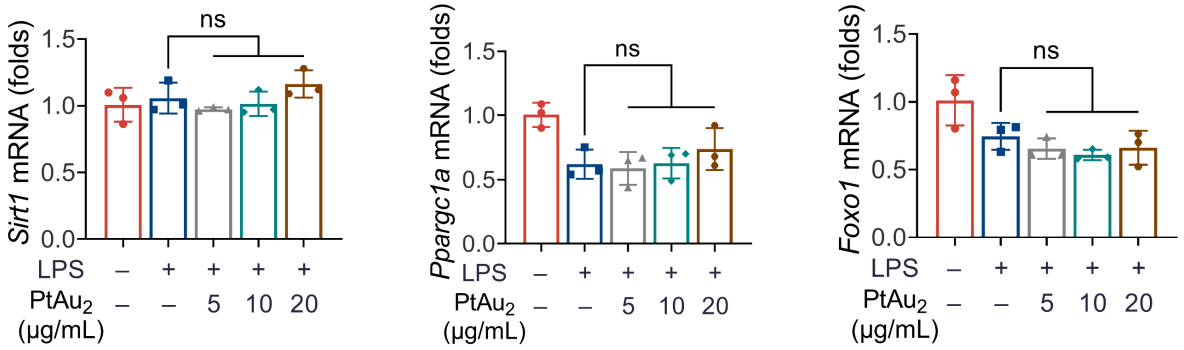

Figure S25. RT-qPCR analysis of gene expression of anti-oxidative signaling molecules in LPS-activated RAW 264.7 macrophages treated with various concentrations of $PtAu_2$ clusters. Data represent the mean ± SD of three independent experiments (one-way ANOVA with Tukey's post hoc test).

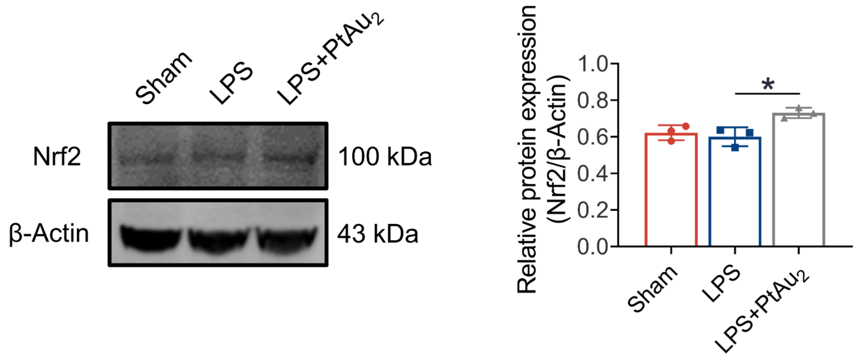

Figure S26. Expression of Nrf2 in mice calvarial tissues of various treatment groups. Data represent the mean ± SD of three independent animals (one-way ANOVA with Tukey's post hoc test). $*p < 0.05$; $**p < 0.01$.

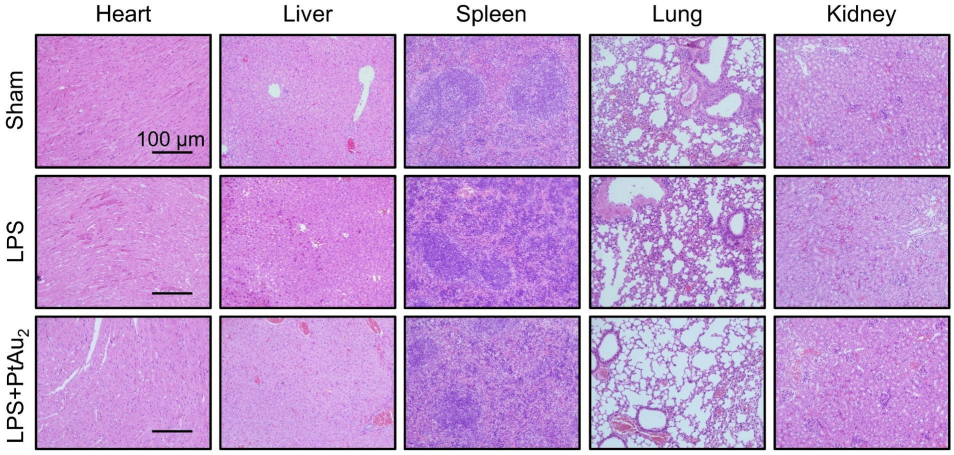

Figure S27. H&E staining images of heart, liver, spleen, lung, and kidney 14 days after model establishment (11 days after $PtAu_2$ clusters treatment).

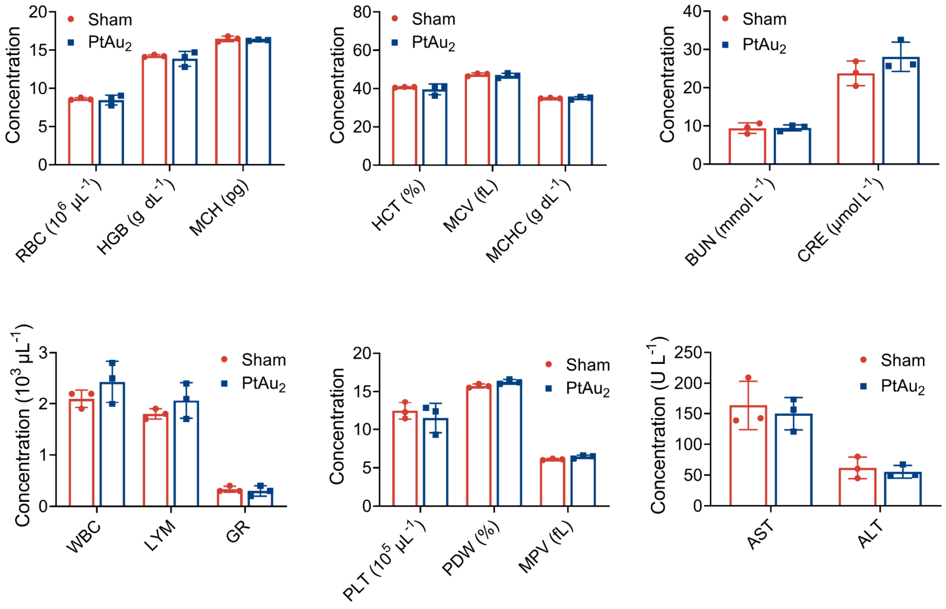

Figure S28. *In vivo* biosafety evaluation of $PtAu_2$ clusters. Complete blood panel analysis and serum biochemistry analysis were performed 14 days after model establishment (11 days after $PtAu_2$ clusters treatment). Data represent the mean ± SD of three independent animals (one-way ANOVA with Tukey's post hoc test). $*p < 0.05$; $**p < 0.01$.

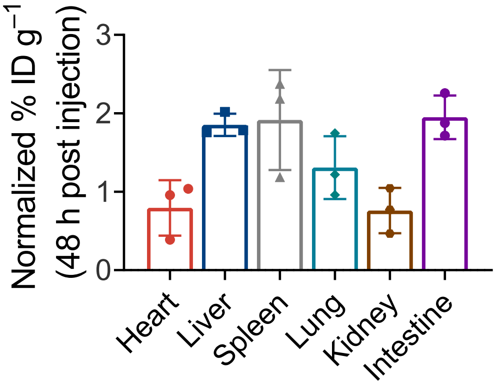

Figure S29. Biodistribution of $PtAu_2$ clusters in major organs at 48 h post injection. Data represent the mean ± SD of three independent animals.

Supplementary Tables

Table S1. Sequences of the RT-qPCR.

Gene	Forward	Reverse
Gapdh	ACCCAGAAGACTGTGGATGG	CACATTGGGGGTAGGAACAC
Tnfα	GCCTCTTCTCATTCCTGCTTGTGG	GTGGTTTGTGAGTGTGAGGGTCT
Il1β	TCGCAGCAGCACATCAACAAGAG	AGGTCCACGGGAAAGACACAGG
Il6	CTTCTTGGGACTGATGCTGGTGAC	AGGTCTGTTGGGAGTGGTATCCTC
Nos2	ACTCAGCCAAGCCCTCACCTAC	TCCAATCTCTGCCTATCCGTCTCG
Nrf2	CAGCCATGACTGATTTAAGCAG	CAGCTGCTTGTTTTCGGTATTA
Hmox1	GAGCAGAACCAGCCTGAACT	AAATCCTGGGGCATGCTGTC
Nqo1	GGTAGCGGCTCCATGTACTC	CGCAGGATGCCACTCTGAAT
Trap	CCATTGTTAGCCACATACGG	CACTCAGCACATAGCCCACA
Ctr	TGCAGACAACTCTTGGTTGG	TCGGTTTCTTCTCCTCTGGA
Dcstamp	AAAACCCTTGGGCTGTTCTT	AATCATGGACGACTCCTTGG
Ctsk	CTTCCAATACGTGCAGCAGA	TCTTCAGGGCTTTCTCGTTC
Sirt1	GCTGACGACTTCGACGACG	TCGGTCAACAGGAGGTTGTCT
Ppargc1	GCACCAGAAAACAGCTCCAAG	CGTCAAACACAGCTTGACAGC
Foxo1	TGTACAGCGCATAGCACCAA	CCGATGGACGGAATGAGAGG

Table S2. Crystallographic Data of $PtAu_2$-**1a** cluster.

Empirical formula	$C_{114}H_{126}Au_2Cl_8F_6N_4O_{14}P_6PtS_2$
Formula weight	3012.74
Crystal system	triclinic
Space group	$P\bar{1}$
a (Å)	12.2198(8)
b (Å)	14.1280(10)
c (Å)	19.5550(14)
α (°)	99.809(3)
β (°)	106.978(2)
γ (°)	94.726(3)
V (Å^3)	3150.2(4)
Z	1
F (000)	1498
ρ_{calcd} (g cm^{-3})	1.588
μ (mm^{-1})	3.777
Radiation (λ, Å)	0.71073
Temperature (K)	293(2)
GOF	1.053
R_1 $(F_o)^a$	0.0292
wR_2 $(F_o^2)^b$	0.0722

^{a} $R1 = \Sigma|F_o - F_c|/\Sigma F_o$, $^b wR2 = \Sigma[w(F_o^2 - F_c^2)^2]/\Sigma[w(F_o)^2)]^{1/2}$

References

1. Chen X, Chen X, Zhou Z, Mao Y, Wang Y, Ma Z, et al. Nirogacestat suppresses RANKL-Induced osteoclast formation in vitro and attenuates LPS-Induced bone resorption in vivo. Exp Cell Res. 2019; 382: 111470.
